# SphK1/S1P signaling-mediated crosstalk between pancreatic acinar cell and macrophage M1 polarization aggravates acute pancreatitis progression

**DOI:** 10.7150/ijbs.120627

**Published:** 2026-03-25

**Authors:** Jie Wang, Guangyu Zhao, Ping Hu, Shengbo Han, Yuhang Hu, Zhu Zeng, Yang Li, Yong Zhao, Yan Huang, Wenfeng Zhuo, Guozheng Lv, Hongda Wang, Gang Zhao

**Affiliations:** Department of Emergency Surgery, Union Hospital, Tongji Medical College, Huazhong University of Science and Technology, Wuhan 430022, China.

**Keywords:** acute pancreatitis, pancreatic acinar cell, macrophages, M1 polarization, SphK1, S1P

## Abstract

Studies have shown that M1 polarization of macrophages plays a crucial role in pathogenesis of acute pancreatitis (AP), although the underlying mechanisms remain incompletely understood. In this study, an *in vivo* AP model was induced in mice using caerulein or L-arginine, while an *in vitro* AP model was established by treating pancreatic acinar cells (PACs) with cholecystokinin (CCK). We observed a significant upregulation of SphK1/S1P in both CCK-treated PACs and the pancreatic tissue of AP mice. In contrast, inflammation and M1 macrophage polarization were markedly attenuated in SphK1^-/-^ AP mice and upon treatment with pharmacological inhibitors targeting SphK1 or S1PR2. Similarly, M1 polarization of macrophages was notably induced by injured pancreatic acinar cells (iPACs), but this effect was suppressed by SphK1 knockdown or inhibition. Mechanistically, S1P derived from iPACs specifically bound to S1PR2 on macrophages, activating PI3K/JNK and ERK pathways to induce M1 polarization. Moreover, TNF-α secreted by M1 macrophages enhanced SphK1 transcription in PACs through NF-κB activation, forming a positive feedback loop between iPACs and macrophage M1 polarization. Collectively, our findings reveal that the SphK1/S1P/S1PR2/TNF-α axis mediates a reciprocal interaction between iPACs and M1 macrophages, which significantly contributes to AP pathogenesis.

## Introduction

Acute pancreatitis (AP) is an inflammatory disorder of the pancreas characterized by acinar cell necrosis and is frequently accompanied by localized or systemically inflammatory responses^1^, ^2^. The annual incidence of AP in developed countries is approximately 34 cases per 100,000 individuals^3^. Although most cases are mild and typically achieve spontaneous resolution within one week, nearly 20% of patients develop moderate or severe phenotype. These advanced forms are marked by pancreatic or peripancreatic necrosis, systemic inflammatory response syndrome (SIRS), and multiple organ dysfunction syndrome (MODS), resulting in a high mortality rate of 20-40%^4^-^7^. Investigations have demonstrated that the initial pathogenic event in AP is damage to PACs, which subsequently triggers the infiltration and activation of macrophages, neutrophils, and other inflammatory cell populations^8^, ^9^. The necrotic pancreatic tissue functions as an inflammatory trigger, thereby stimulating the activation of these immune cells^10^. Among these cells, macrophages are one of the earliest populations to infiltrate the pancreas during AP. While their physiological roles involve host defense and clearance of cellular debris and necrotic material, their activation paradoxically exacerbates inflammation and contributes to AP progression^11^. Consequently, elucidating the mechanisms that govern macrophage infiltration and activation is critical for the development of effective therapeutic strategies for AP.

Macrophages are a critical component of the immune system, playing diverse roles in inflammation, tissue repair, and host immunity^12^, ^13^. The classically activated M1 phenotype, induced by interferon-gamma (IFN-γ) or lipopolysaccharide (LPS), is closely associated with antibacterial response and inflammatory processes. This subtype is characterized by the production of pro-inflammatory cytokines that initiate and amplify immune reactions^14^. In the pancreatic microenvironment, macrophages constitute the predominant infiltrating immune cell population. Their activation results in the robust secretion of inflammatory mediators, thereby amplifying the local inflammatory cascade^15^, ^16^. Recent studies have revealed that macrophages within necrotic pancreatic tissue can phagocytose zymogen granules released from iPACs. This process can induce zymogen activation through a tissue histone B-dependent pathway intrinsic to macrophages, leading to enhanced inflammatory response and exacerbation of pancreatitis severity^17^. Therefore, these findings suggest that macrophages polarization is a key regulatory factor in AP progression, but the regulatory mechanism governing macrophages polarization remain to be fully elucidated.

SphK1 is a key rate-limiting enzyme in sphingolipid metabolism that phosphorylates sphingosine to produce S1P^18^. S1P subsequently binds to its cognate receptors (S1PRs), tirggering intracellular signaling cascades through autocrine or paracrine manner^19^. The SphK1-S1P-S1PRs signaling axis has been implicated in the pathogenesis of numerous inflammatory disorders^20^-^22^. In SAP, previous studies have reported increased expression and activity of SphK1 in peripheral neutrophils, monocytes, and lymphocytes, and that SphK1 may modulate the inflammatory responses through S1PR3 signaling^23^. Furthermore, recent work in chronic pancreatitis has showed that activation of the SphK1/S1P axis in PACs promoted pancreatic fibrosis by inducing autophagy and activating pancreatic stellate cells via the AMPK/mTOR pathway^24^.

In this study, we first examined SphK1 expression and subsequently employed SphK1 knockout mice to evaluate its effects on inflammation and macrophage M1 polarization. To further unravel the regulatory role of SphK1 in AP, we developed a co-culture system of PACs and macrophages to study their interplay. Additionally, we assessed the therapeutic effects of both SphK1 and S1PR2 inhibitors in AP mice. Our research highlight that the interaction between iPACs and macrophage M1 polarization, mediated through the SphK1/S1P/S1PR2 signaling axis, plays a critical role in AP pathogenesis and may present a potential therapeutic target.

## Materials and Methods

### Animals

To induce AP model, 6-8 weeks old female mice were fasted for 12 hours and randomly assigned to groups (n = 5 per group). For the caerulein-induced AP model, mice received 7 hourly intraperitoneal injections of caerulein (50 ug/kg). Serum and pancreatic tissue samples were collected at 12 hours or at specified time points. For the L-arginine-induced AP model, mice received 2 hourly intraperitoneal injections of 10% L-arginine (3 g/kg) and were euthanized at 72 hours post-injection. For SphK1 and S1PR2 inhibitor treatment, mice received daily intraperitoneally injections of 10 mg/kg PF-543 (MedChemExpress, Monmouth Junction, NJ) and 30 mg/kg JTE-013 (MedChemExpress, Monmouth Junction, NJ), beginning 1 day after the initial caerulein or L-arginine injection. The SphK1 knockout mice used in this study were generously provided by Professor Zhou Hong (Anhui Medical University). All animal experiments were approved by the Institutional Animal Care and Use Committee of Huazhong University of Science and Technology (approval no. IACUC 3618) and were conducted in accordance with the National Institutes of Health guidelines for the care and use of laboratory animals.

### Histology, immunohistochemistry, and immunofluorescence

Mice were euthanized by cervical dislocation. Pancreatic tissues were harvested, formalin-fixed, paraffin-embedded, and sectioned (4 µm). Tissue sections were subjected to hematoxylin and eosin (H&E) staining, immunohistochemistry, and immunofluorescence. The severity of pancreatitis and histopathological scores were evaluated as previously described^25^.

Immunohistochemistry was performed according to the manufacturer's instructions: tissue sections underwent antigen retrieval using EDTA buffer, followed by incubation with the indicated primary antibodies at 4 ℃ for 24 hours in the dark. Visualization was achieved using DAB, and nucleus were counterstained with hematoxylin. Finally, sections were then dehydrated and mounted with neutral balsam for pathology evaluation. For immunofluorescence staining, pancreatic tissue sections were fixed with methanol and incubated with an anti-SphK1 antibody (PA5-14068, Thermo Fisher Scientific) at a dilution of 1:250 in BSA. Macrophages were labeled using an anti-F4/80 antibody (Cell Signaling Technology, 70076), and M1 macrophages were identified using an anti-CD86 antibody (Cell Signaling Technology, 19589).

### Isolation of bone marrow derived macrophages (BMDMs)

Bone marrow cells were isolated by flushing the femur and tibia with phosphate-buffered saline and passing the cell suspension through a 100-mm cell strainer. Cells were collected in a 50 mL tube, centrifuged, and erythrocytes were removed. BMDMs were then cultured in medium supplemented with GM-CSF. The culture medium was refreshed every 2 days. By day 6, cells had differentiated into M0 macrophages and were used for subsequent experiments.

### Cell treatments

266-6 and RAW264.7 cells were cultured in DMEM supplemented with 10% fetal bovine serum (Gibco, USA) at 37 °C in a humidified incubator 5% CO_2_. Cells were transfected with specific or control siRNAs (GenePharma) using Lipofectamine 3000 (Invitrogen) according to the manufacturer's instructions. To evaluate SphK1 and S1P expression, 266-6 cells were treated with 400nM cholecystokinin (CCK, Absin) or DMSO as a control. For inhibition of SphK1 activity, 266-6 cells were treated with 10 μM PF-543 (MedChemExpress) for 24 hours. To investigate the role of S1P in macrophages, RAW264.7 cells were treated with 1 μM S1P (Sigma) and 1 μM JTE-013 (MedChemExpress). Conditioned media (CM) was collected from cultured 266-6 cells, centrifuged to remove cell debris, and the supernatant was filtered through a 70 mm filter before storage at -80 °C. Prior to use, CM was diluted 1:1 with DMEM containing 10% FBS.

### Transfection

SphK1 siRNA (siSphK1), S1PR2 siRNA (siS1PR2), and negative control siRNAs (siNC) were purchased from GenePharma Co. (Suzhou, China). For siRNA transfection, HiPerFect (QIAGEN) or Lipofectamine 3000 (Invitrogen, USA) was used according to the manufacturers' instructions, with Opti-MEM I (Invitrogen, USA) as the transfection medium. The sequences of the siRNAs and vectors are provided in Supplementary Table 1.

### Real-time fluorescent quantitative polymerase chain reaction

Total RNA was extracted from mouse pancreatic tissues or cultured cells using RNAiso Plus reagent (TaKaRa Bio, Japan) and quantified with a NanoDrop spectrophotometer (K5600, KAIAO). The RNA was then reverse transcribed into cDNAs using a reverse transcription kit according to the manufacturer's instructions. Quantitative polymerase chain reaction (qPCR) was performed using a StepOnePlus^TM^ PCR system (Applied Biosystems, USA). Primer sequences used for qPCR are provided in Supplementary Table 2.

### Western blot assay analysis

Total proteins from cells or tissues were extracted using lysis buffer (Beyotime) supplemented with proteinase inhibitors (MedChemExpres). Proteins samples were separated by sodium dodecyl sulfate-polyacrylamide gel electrophoresis (SDS-PAGE) and transferred onto nitrocellulose membranes (Millipore). After blocking in 5% skim milk, membranes were incubated overnight at 4 °C with the following primary antibodies: SphK1 (PA5-14068, Thermo Fisher Scientific), CD86 (13395-1-AP, Proteintech), iNOS(22226-1-AP, Proteintech), NF-κB p65 (10745-1-AP, Proteintech), phospho-p65 (ab76302, abcam), S1PR2 (21180-1-AP, Proteintech), PI3K (4249, Cell Signaling TECHNOLOGY), phospho-PI3K (17366, Cell Signaling TECHNOLOGY), JNK (9252, Cell Signaling TECHNOLOGY), phospho-JNK (4668, Cell Signaling TECHNOLOGY), and ERK (11257-1-AP, Proteintech), and phospho-ERK (28733-1-AP, Proteintech). Membranes were then incubated with horseradish peroxidase-conjugated secondary antibodies for 1 hour at room temperature. Finally, immunoreactive bands were detected using an ultrasensitive ECL Chemiluminescence Kit (BioSharp), and images were captured with Image Lab 6.1 software (Bio-Rad).

### Luciferase activity assay

266-6 cells expressing the pGL3-based reporter construct containing the SphK1 HRE were transfected with siNF-κB or siNC. After 24 hours of transfection, cells were cultured for an additional 24 hours. Luciferase activity was measured using the luciferase assay kit (GenePharma) and plotted after normalizing with respect to Renilla luciferase activity. Firefly luciferase activity was measured using the Dual-Luciferase Reporter Assay System (Promega, Madison, WI, USA) and normalized to Renilla luciferase activity. All experiments were performed independently in triplicate, with six samples per group.

### Flow cytometry

RAW264.7 cells were harvested and prepared as single-cell suspensions, then incubated with anti-CD86 antibodies (BioLegend) for 30 minutes at 4 °C in the dark. After two washes, cells were resuspended in 500 μL of phosphate-buffered saline (PBS) and analyzed on a FACSCanto TMII cytometer (BD Biosciences). All the tests were controlled by the homologous isotype control antibodies. Data were analyzed using FlowJo software (BD Biosciences).

### Chromatin immunoprecipitation

The chromatin immunoprecipitation (CHIP) assay was performed according to the manufacturer's instructions using the EZ-ChIP^TM^ kit (Millipore, MA, USA). 266-6 cells were seeded in 15-cm dishes and cross-linked with 1% formaldehyde for 20 minutes. Cells were then lysated, and chromatin was sonicated to generate DNA fragments of approximately 100-200 bp. The lysates were incubated with specific antibodies to form DNA-protein complexes. After washing, the DNA fragments was analyzed by RT-qPCR. Normal rabbit IgG antibody (Proteintech, 30000-0-AP) was used as a negative control.

### ELISA assay

ELISA was conducted following the manufacturer's instructions. Human and mouse serum samples were obtained by centrifugation of whole blood at 4 °C for 20 minutes, and the supernatants were collected for subsequent analysis. First, the reaction wells were closed with 5% fetal bovine serum for 40 minutes. Then, the blank, negative, and positive control wells were prepared, followed by the addition of 100 μl of freshly diluted enzyme-conjugated secondary antibody. The plates were incubated at 37 °C for 60 minutes and then washed. TMB substrate solution was added to each well and incubated at 37 °C for 5-10 minutes, after which the reaction was stopped by adding 50 μl of stop buffer. Finally, absorbance was measured at 450nm using microplate reader. S1P concentration in mouse serum, human serum, and cell culture supernatants were quantified using a commercial ELISA kit (Cat. HYYX200451, HYCEZMBIO®, China).

### Trypsin activity assay

Peripheral blood and pancreatic tissue samples were collected from mice euthanized with pentobarbital. Serum amylase and lipase levels were measured using enzymatic assays kits (Nanjing Jiancheng Bioengineering Institute) according to the manufacturer's instructions.

### Macrophage depletion

Macrophages were depleted using sodium clodronate liposomes (Encapsula NanoSciences, USA): Mice received an intraperitoneal injection of clodronate liposomes (200 μL per mouse) or control PBS liposomes, followed by a second intraperitoneal dose (100 μL) 2 days later. 48 hours after the second injection, the efficiency of macrophage depletion was evaluated by immunohistochemical staining. Exogenous S1P (5 mg/kg) was administered via intravenous injection after AP induction.

### Statistical analysis

Statistical analyses were performed using GraphPad Prism (GraphPad Software, USA). Data are presented as mean ± standard deviation (SD). The sample size (n) for each experiment is indicated in the corresponding figure legends and represents independent biological replicates. For animal experiments, each mouse was considered an independent experimental unit. All experiments were performed at least three times independently. Comparisons between two groups were conducted using unpaired Student's *t*-test. For multiple-group comparisons, one-way ANOVA followed by Tukey's post hoc test was applied. Exact P-values are provided where applicable, and a *P*-value < 0.05 was considered statistically significant.

## Results

### SphK1 is overexpressed in the PACs of AP mice

**Figure 1:**
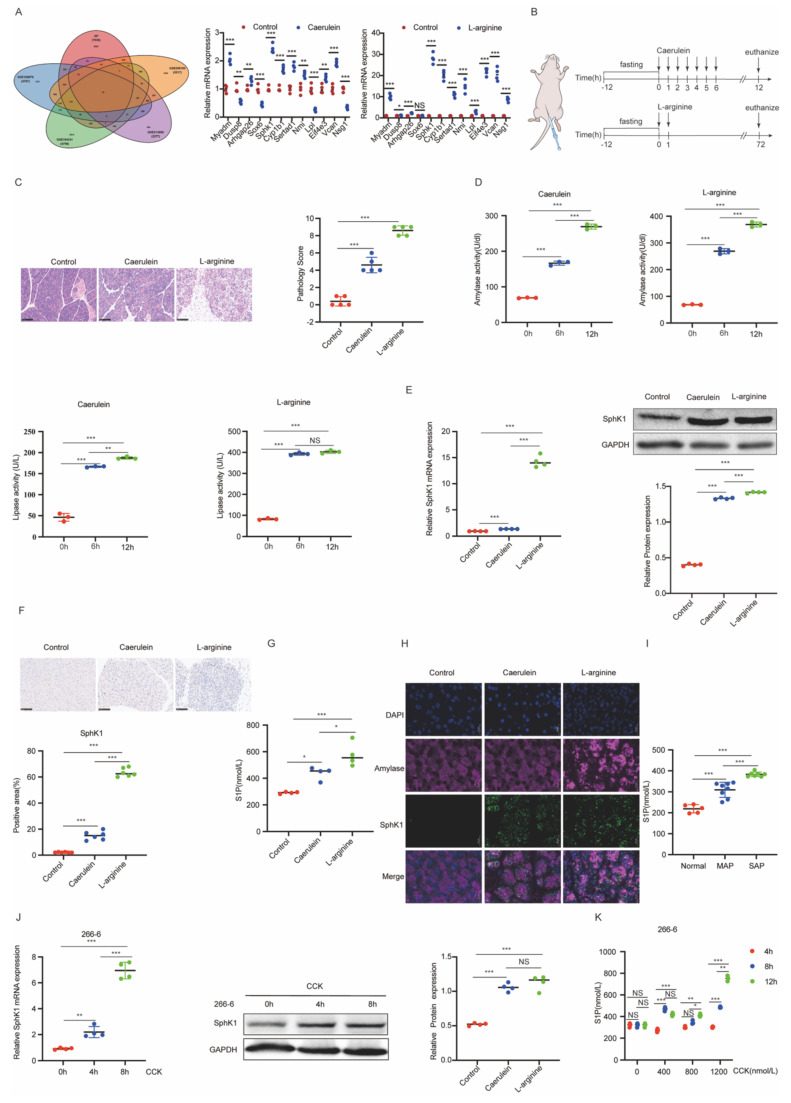
** SphK1 is overexpressed in the PACs of AP mice.** (A) Venn diagram showing the overlap of differentially expressed genes associated with AP, identified from RNA-sequencing data of CCK-treated 266-6 cells and datasets from the GEO database. (B) Generation of a caerulein or L-arginine induced AP model. (C) Representative images of H&E staining in the pancreatic tissues of AP mice (n=5). Scale bars, 100 μm. (D) Pancreatic exocrine function was evaluated by measuring serum AMY and LPS levels. (E) The mRNA and protein levels of SphK1 in pancreatic tissues were evaluated by RT-qPCR and Western blot in AP mice. (F) Representative images of IHC staining for SphK1 in the pancreatic tissues of AP mice (n=6). Scale bars, 100 μm. (G) The level of S1P in mouse serum was measured by ELISA (n=4). (H) The expression of SphK1 (in green) and Amylase (in red) was detected by tissue immunofluorescence double localization. Nuclei were counterstained with DAPI (in blue). Scale bar: 50 μm. (I) The level of S1P in AP patients was measured by ELISA (n=5). (J) The mRNA and protein levels of SphK1 in CCK-treated 266-6 cells were evaluated by RT-qPCR and Western blot. (K) The S1P level in the culture media of CCK-treated 266-6 cells was measured by ELISA. *P <0.05; **P <0.01; ***P <0.001.

To identify potential pro-inflammatory genes associated with AP, we first analyzed RNA-seq data from CCK-treated 266-6 cells, together with publicly available datasets from GEO database (GSE169076, GSE194331 GSE214666, GSE298193), which included caerulein-induced and L-arginine induced AP mice models as well as peripheral blood samples from AP patients. The integrated analysis consistently revealed upregulation of SphK1 in AP mice tissues, AP patients' samples, and CCK-treated 266-6 cells (Figure. 1A). To further confirm SphK1 upregulation in AP, we developed caerulein and L-arginine induced mouse models of AP (Figure 1B). H&E staining confirmed successful induction of AP, as evidenced by pancreatic edema, inflammatory cell infiltration, and acinar cell necrosis in AP mice (Figure 1C). Correspondingly, serum amylase and lipase levels were also elevated (Figure 1D). RT-qPCR, Western Blot, and immunohistochemical (IHC) all confirmed significant upregulation of SphK1 in pancreatic tissues from AP mice (Figure 1E and 1F). In addition, serum S1P levels were significantly increased in AP mice (Figure 1G).

Immunofluorescence (IF) double staining showed SphK1 predominantly localized to acinar regions in pancreatic tissue from AP mouse (Figure 1H). We also found elevated S1P levels in plasma samples from AP patients (Figure 1I). *In vitro*, both SphK1 expression and S1P production were significantly increased in CCK-treated 266-6 cells (Figure 1J and 1K). Collectively, these findings indicate that SphK1/S1P signaling is upregulated in acinar cells during AP, suggesting a potential role in AP progression.

### Knockdown of SphK1 mitigates the severity of AP with decreased macrophage M1 polarization

**Figure 2:**
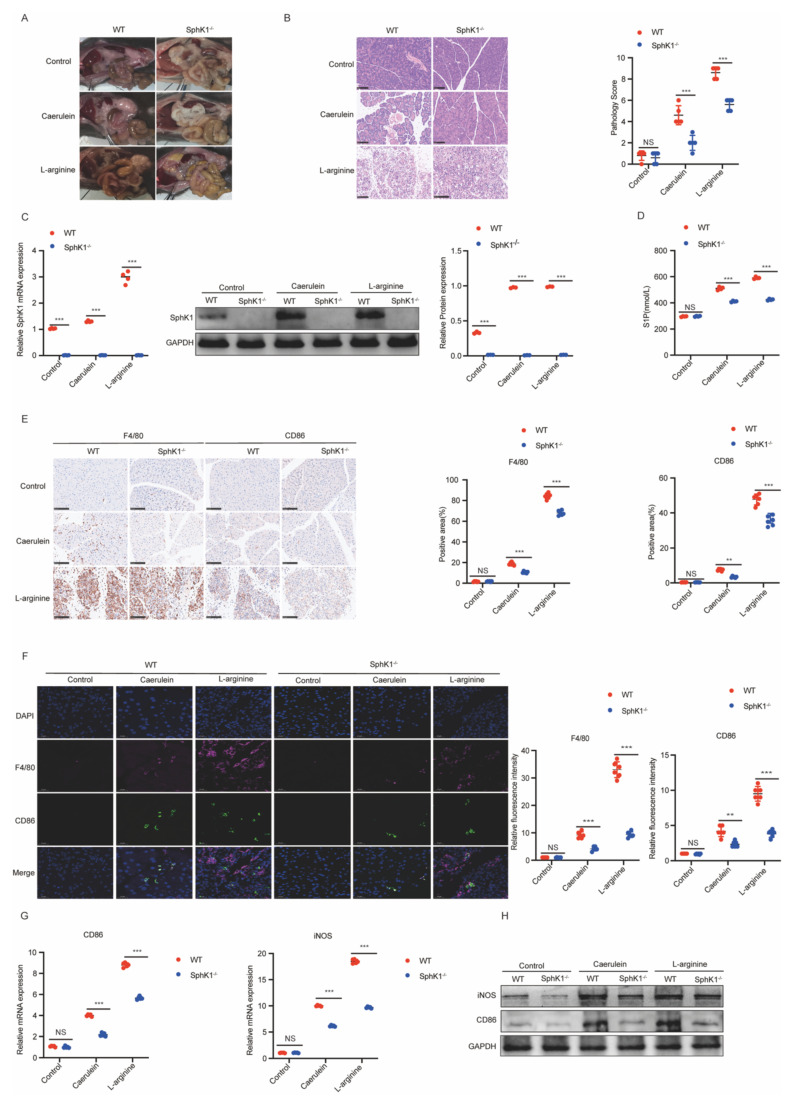
** Knockdown of SphK1 mitigates the severity of AP with decreased macrophage M1 polarization.** AP was induced in WT and SphK1^-/-^ mice by caerulein and L-arginine, respectively. (A) Representative images of the pancreatic tissues in WT and SphK1^-/-^ AP mice on naked eye. (B) Representative images of H&E staining in the pancreatic tissues of WT and SphK1^-/-^ AP mice (n=5). Scale bar: 100 μm. (C) RT-qPCR and Western blot analysis of SphK1 expression in the pancreatic tissues of WT and SphK1^-/-^ AP mice. (D) The S1P levels in the serum of WT and SphK1^-/-^ AP mice. (E) The expression of F4/80, and CD86 in the pancreatic tissues of WT and SphK1^-/-^ AP mice were evaluated by IHC (n=6). Scale bar: 100 μm. (F) The expression of F4/80 and CD86 in the pancreatic tissues of WT and SphK1^-/-^ AP mice were evaluated by IF (n=6). Scale bar: 20 μm. (G) The mRNA expression of CD86 and iNOS in the pancreatic tissues of WT and SphK1^-/-^ AP mice. (H) The protein expression of CD86 and iNOS in the pancreatic tissues of WT and SphK1^-/-^ AP mice. *P <0.05; **P <0.01; ***P <0.001.

Given the upregulation of SphK1 in AP, we next explored its functional role. SphK1 knockout (SphK1^-/-^) mice exhibited markedly reduced pancreatic injury and inflammation compared to wild-type (WT) mice in both caerulein- and L-arginine-induced AP models, as demonstrated by H&E staining (Figure 2A and 2B). The absence of SphK1 expression in the pancreatic tissues from SphK1^-/-^ AP mice was confirmed by RT-qPCR and Western Blot analyses (Figure 2C). Additionally, serum amylase and lipase levels were significantly lower in SphK1^-/-^ AP mice (Supplementary Figure 1A). Consistently, serum S1P levels were markedly reduced in these mice (Figure 2D).

Considering the established role of M1 macrophage polarization in AP progression, we next investigated whether SphK1/S1P signaling contributes to this process. IHC and IF double staining for F4/80 (a macrophage marker) and CD86 (an M1 polarization marker) revealed a marked increase in M1-polarized macrophages in both caerulein- and L-arginine-induced AP models in WT mice (Supplementary Figure 1B and 1C). Importantly, this increase was significantly attenuated in SphK1^-/-^ AP mice (Figure 2E and 2F). Similarly, the expression levels of CD86 and iNOS in pancreatic tissues were markedly decreased in SphK1^-/-^ AP mice (Figure 2G and 2H). Collectively, these findings suggest that SphK1/S1P signaling contributes to AP progression by promoting macrophage M1 polarization.

### SphK1/S1P signaling is required for iPAC-induced M1 polarization of macrophages

The dynamic interaction between PACs and macrophages is a key driver of AP progression^26^, ^27^. Given that S1P has been reported to promote M1 polarization of macrophages in various inflammatory conditions^28^, ^29^, and considering our observation of upregulated SphK1 and S1P in AP mice, we hypothesized that SphK1-mediated S1P release from iPACs promotes M1 polarization of macrophages in AP.

**Figure 3:**
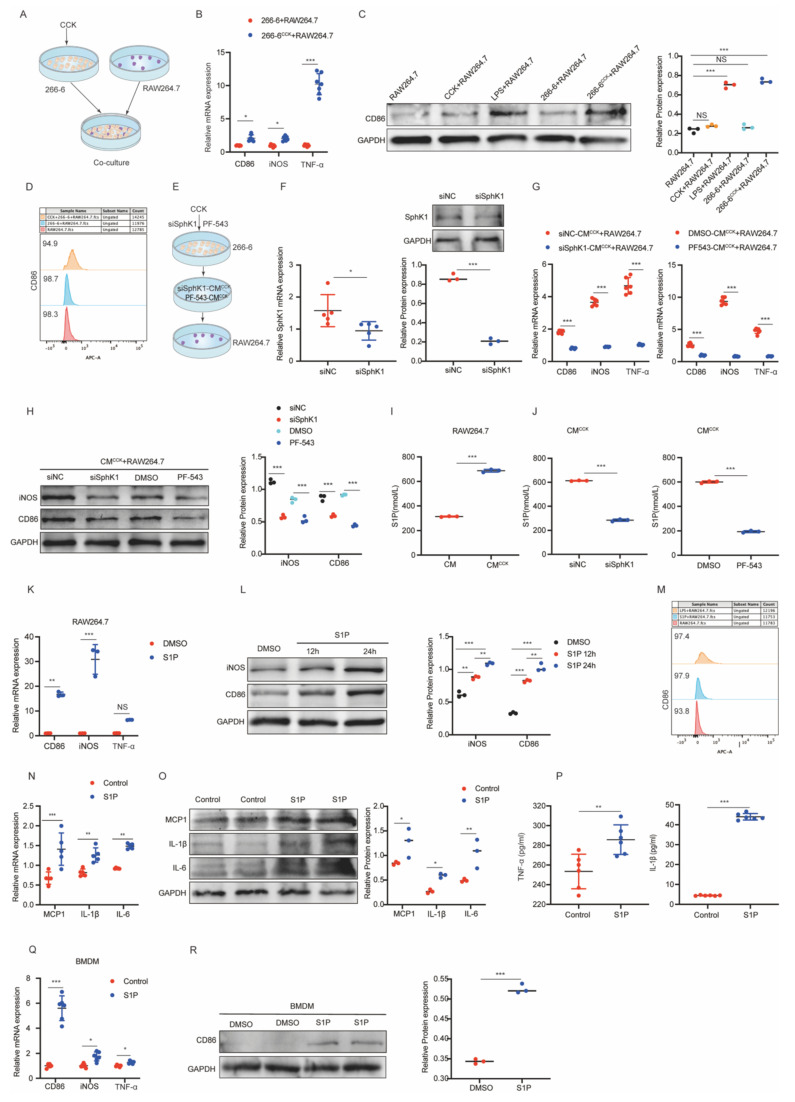
** SphK1/S1P signaling is required for iPAC-induced M1 polarization of macrophages.** 266-6 cells were treated with CCK to simulate the injured PACs (iPACs) in AP. (A) Schematics of the co-culture system involving CCK-treated 266-6 cells and RAW264.7 cells. (B) The mRNA level of CD86, iNOS, and TNF-α in RAW264.7 cells co-cultured with CCK-injured 266-6 cells. (C) The protein level of CD86 in RAW264.7 cells co-cultured with CCK-injured 266-6 cells. (D) The positive cells of CD86 in RAW264.7 cells following co-cultured with CCK-injured 266-6 cells was assessed by flow cytometry. (E-H) The mRNA level of CD86, iNOS, and TNF-α and the protein level of CD86 and iNOS in CM treated RAW264.7 cells when SphK1 was knockdown by siRNA or PF-543. (I) The content of S1P in RAW264.7 cells supernatant following co-cultured with CM^CCK^ was determined by ELISA. (J) The S1P level in RAW264.7 cells co-cultured with pretreated with siSphK1 or PF-543 CM^CCK^. (K) The mRNA level of CD86, iNOS, and TNF-α in S1P-treated RAW264.7 cells. (L) The protein level of CD86 and iNOS in S1P-treated RAW264.7 cells. (M) The positive cells of CD86 in S1P-treated RAW264.7 cells by flow cytometry. (N-O) The mRNA and protein levels of MCP1, IL-1β, and IL-6 in S1P-treated RAW264.7 cells. (P) The level of TNF-α and IL-1β in the supernatant of S1P-treated RAW264.7 cells. (Q-R) The mRNA level of CD86, iNOS, and TNF-α and the protein level of CD86 and iNOS in S1P-treated BMDMs. *P <0.05; **P <0.01; ***P <0.001; NS, no significance.

To test this hypothesis, RAW264.7 cells were co-cultured with CCK-pretreated 266-6 cells (Figure 3A). Co-culture with CCK-treated 266-6 cells significantly increased the expression of M1 polarization markers, including CD86, iNOS, TNF-α, in RAW264.7 cells (Figure 3B-3D). Consistently, treatment of RAW264.7 cells with conditioned media (CM) derived from CCK-treated 266-6 cells (CM^CCK^) produced similar effects (Supplementary Figure 2A-2C) and markedly elevated the expression of proinflammatory cytokines, including TNF-α, IL-1β, IL-6 and MCP1 (Supplementary Figure 2D-2F). Importantly, these findings were further validated using injured primary acinar cells, which similarly promoted macrophage M1 polarization (Supplementary Figure 8A-C), thereby confirming the credibility of the conclusions. Furthermore, inhibition of SphK1 in 266-6 cells with siSphK1 or PF-543 remarkably blocked CM^CCK^-induce M1 polarization in RAW264.7 cells (Figure 3E-H). Additionally, S1P levels were significantly elevated in CM^CCK^, and that this increase was dependent on SphK1 activity (Figure 3I and 3J). Notably, CCK treatment did not directly affect SphK1/S1P expression or secretion in macrophages (Supplementary Figure 2G-2I).

To directly evaluate the role of S1P, macrophages were treated with exogenous S1P. Exogenous S1P stimulation significantly increased the expression of M1 markers (CD86, iNOS) and elevated the proportion of CD86-positive cells in RAW264.7 cells (Figure 3K-3M). Additionally, S1P markedly upregulated TNF-α, IL-1β, IL-6, and MCP1 expression (Figure 3N-3P). Consistently, primary macrophages derived from bone marrow (BMDSs) exposed to exogenous S1P also showed elevated levels of CD86, iNOS and TNF-α (Figure 3Q and 3R). Furthermore, S1P supplementation restored M1 polarization in RAW264.7 cells treated with CM^CCK^ from SphK1-inhibited 266-6 cells (Supplementary Figure 2J and 2K). Collectively, these data support that SphK1-S1P pathway in iPACs plays a pivotal role in driving macrophages M1 polarization during AP.

### S1PR2 is necessary for S1P-induced M1 macrophages polarization

**Figure 4:**
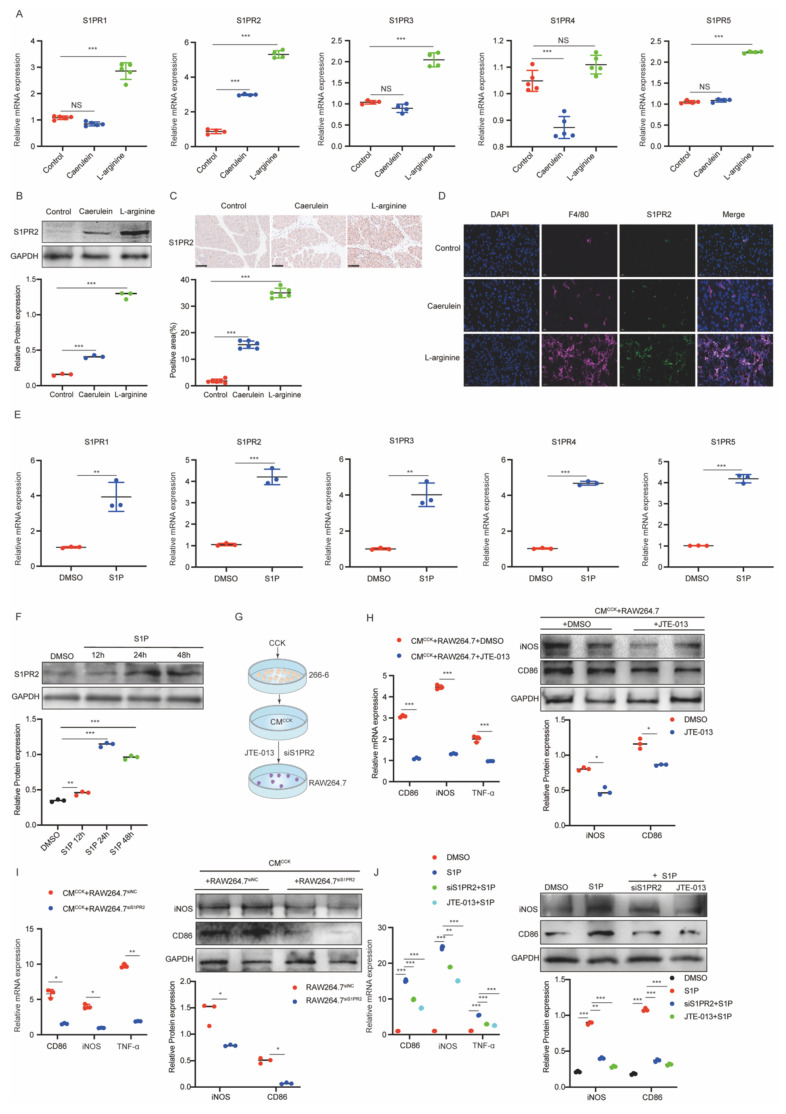
** S1PR2 is necessary for S1P-induced M1 macrophages polarization.** (A) RT-qPCR was performed to measure the mRNA levels of S1PRs in pancreatic tissues from AP mice. (B-C) Western blot and IHC analysis of S1PR2 expression in pancreatic tissues from AP mice. (D) IF images of AP mice pancreatic tissues stained with F4/80(in red) and S1PR2 (in green). Nuclei were counterstained with DAPI (in blue). Scale bar: 20 μm. (E) The mRNA levels of S1PRs in RAW264.7 cells following S1P treatment. (F) Western blot analysis of S1PR2 expression in S1P-treated RAW264.7 cells. (G-I) The mRNA levels of CD86, iNOS, and TNF-α and the protein level of CD86 and iNOS in RAW264.7 cells treated with CM^CCK^ after transfection of siS1PR2 or addition of JTE-013. (J) When exogenous S1P was added, the mRNA and protein levels of CD86, iNOS, and TNF-α in RAW264.7 cells treated with CM^CCK^ after transfection of siS1PR2 or addition of JTE-013. *P <0.05; **P <0.01; ***P <0.001; NS, no significance.

We confirmed that iPACs-derived S1P was upregulated and required for macrophage M1 polarization. However, the specific S1P receptor mediating this process in AP remained unknown. To identify the receptor involved, we first examined the expression of S1PR1-5 in AP mice. S1PR2 exhibited the most pronounced upregulation at both the mRNA (Figure 4A) and protein (Figure 4B) levels compared to other S1PRs. IHC analysis further revealed a significant increase of S1PR2 in pancreatic tissue from AP mice (Figure 4C). Specifically, IF double staining for S1PR2 with F4/80 showed that S1PR2 was predominantly localized in pancreatic macrophages in AP mice (Figure 4D). We next assessed S1PR expression in macrophages treated with S1P. Although mRNAs levels of all S1PRs (S1PR1-5) were elevated in S1P-treated RAW264.7 cells, S1PR2 exhibited the most significant increase (Figure 4E and Supplementary Figure 3A). Consistently, S1PR2 protein expression increased in a time-dependent manner following S1P stimulation (Figure 4F), which was further confirmed by IF staining. Moreover, S1PR2 was primarily localized to the macrophage membrane (Supplementary Figure 3B). To further elucidate the role of S1PR2 in S1P-induced M1 polarization, S1PR2 was silenced using siS1PR2 or inhibited with JTE-013 (Figure 4G, Supplementary Figure 3C). Both interventions significantly attenuated CM^CCK^-induced M1 polarization in RAW264.7 cells (Figure 4H and 4I). Similarly, S1P-induced M1 polarization was markedly suppressed by siS1PR2 or JTE-013 pretreatment (Figure 4J). Collectively, these findings suggest that S1PR2 acts as the primary macrophage receptor mediating S1P-induced M1 polarization during AP.

### S1P/S1PR2 signaling induces M1 polarization in macrophages by activating PI3K/JNK and ERK pathways

**Figure 5:**
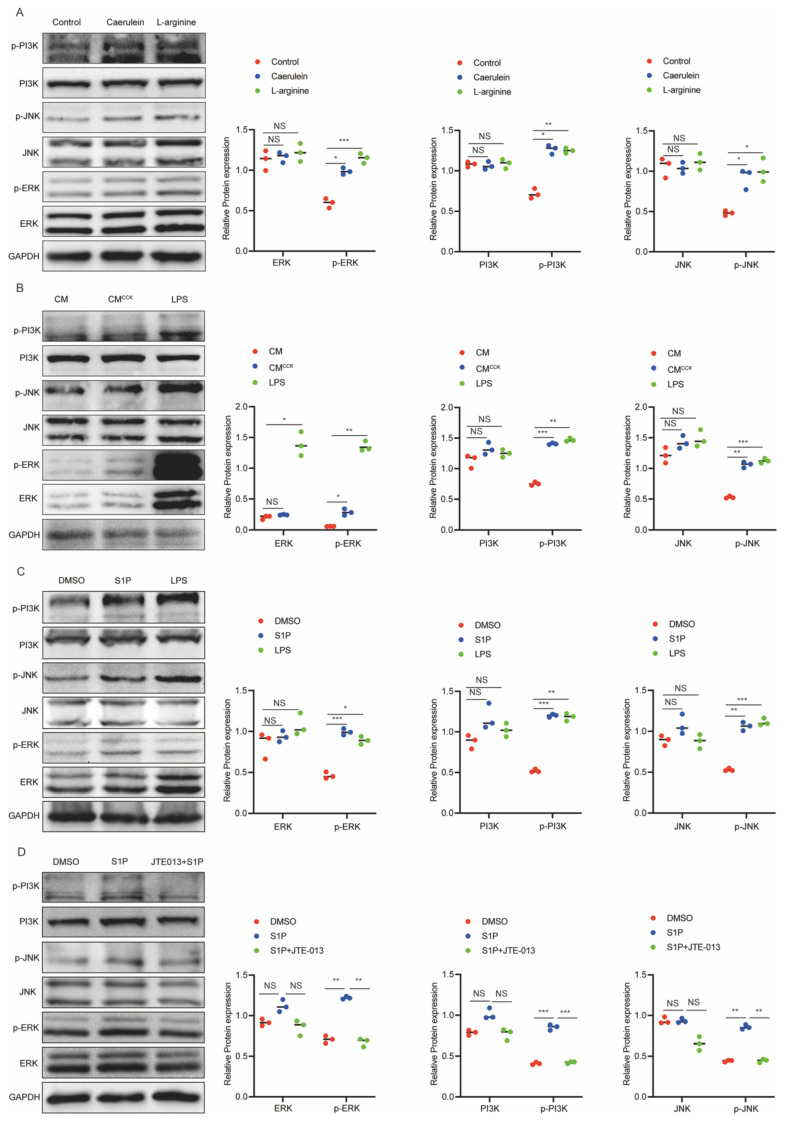
** S1P/S1PR2 signaling induces M1 polarization in macrophages by activating PI3K/JNK and ERK pathways.** (A) Protein levels of PI3K, JNK and ERK in pancreatic tissues from AP mice were analyzed by Western blot. (B) Protein levels of PI3K, JNK and ERK in RAW264.7 cells after treatment with CM^CCK^ or LPS were analyzed by western blot. (C) Protein levels of PI3K, JNK and ERK in S1P-treated RAW264.7 cells were analyzed by western blot. (D) Protein levels of PI3K, JNK and ERK in S1P-treated RAW264.7 cells were assessed by western blot following JTE-013 addition. *P <0.05; **P <0.01; ***P <0.001; NS, no significance.

S1P elicits diverse cellular responses through binding to S1PRs^30^, and has been reported to activate downstream signaling pathways such as the PI3K/AKT^31^, ^32^. Concurrently, previous studies have implicated the PI3K/AKT/JNK and MAPK/ERK pathways in regulating M1 macrophage polarization under various pathological conditions^33^-^35^. To explore these relevant signaling mechanisms, we specifically examined the activation status of the PI3K, JNK, ERK, and AKT pathways. Our results showed that the phosphorylation levels of PI3K, JNK, and ERK were significantly increased in pancreatic tissue from AP mice (Figure 5A). Consistently, similar to LPS stimulation, CM^CCK^ and S1P markedly enhanced the phosphorylation of PI3K, JNK, and ERK in RAW264.7 cells (Figure 5B and C). Moreover, pretreatment with JTE-013 effectively suppressed S1P-induced phosphorylation of these signaling molecules (Figure 5D). These findings suggest that S1P drives M1 macrophage polarization primarily through activation of the PI3K/JNK and ERK pathways.

### M1-polarized macrophages enhance SphK1 expression in PACs through the TNF-α/NF-κB pathway

**Figure 6:**
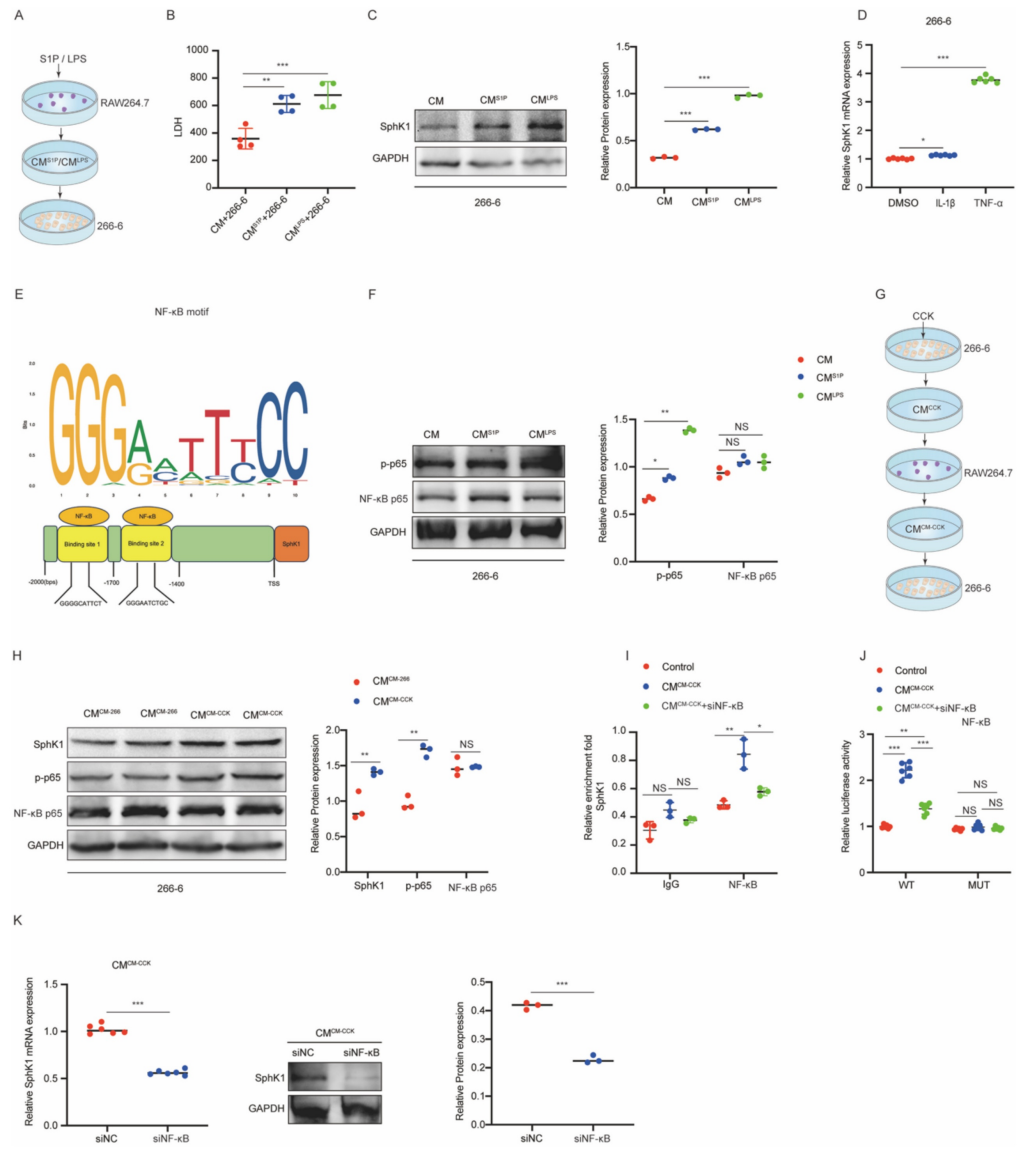
** M1-polarized macrophages enhance SphK1 expression in PACs through the TNF-α/NF-κB pathway.** (A-B) LDH level in the culture media of 266-6 cells treated with CM^S1P^ or CM^LPS^. (C) Western blot analysis of SphK1 expression in CM^S1P^ or CM^LPS^-treated 266-6 cells. (D) The mRNA level of SphK1 in 266-6 cells under the treatment of TNF-α or IL-1β. (E) Schematic illustration of the NF-κB in the promoter of SphK1. (F) The protein level of NF-κB p65 and p-p65 in CM^S1P^ or CM^LPS^-treated 266-6 cells. (G-H) The protein level of SphK1, NF-κB p65 and p-p65 in 266-6 cells pretreated with CM^CM-CCK^. (I) ChIP assays with anti-NF-κB antibody verifying the binding of NF-κB in the SphK1 promoter, and with knockdown of NF-κB. (J) 266-6 cells were transfected with pGL3 reporter vector containing WT or MUT SphK1 promoter. Luciferase activity was measured using the dual-luciferase reporter assay system. (K) The mRNA and protein levels of SphK1 were determined when NF-κB was knockdown in 266-6 cells. *P <0.05; **P <0.01; ***P <0.001; NS, no significance.

To further investigate the reciprocal interactions between M1-polarized macrophages and PACs, 266-6 cells were treated with CM from RAW264.7 cells stimulated with S1P or LPS (Figure 6A). Treatment with CM^S1P^ or CM^LPS^ obviously increased LDH release and SphK1 expression in 266-6 cells (Figure 6B and 6C), indicating that M1-polarized macrophages exacerbated PACs injury and induced SphK1 upregulation, thereby suggesting the presence of a potential feedback loop between iPACs and macrophages.

Our previous work demonstrated TNF-α and IL-1β levels were elevated during M1 polarization. To determine whether these cytokines induce SphK1 expression, 266-6 cells were treated with recombinant TNF-α or IL-1β. RT-qPCR analysis showed a significant increase in SphK1 mRNA level following TNF-α treatment, but not IL-1β (Figure 6D), suggesting that SphK1 was transcriptionally regulated by TNF-α. Using the JASPAR database (https://jaspar.elixir.no/), NF-κB binding sites were predicted within the SphK1 promoter region (Figure 6E). Furthermore, treatment with CM^S1P^ or CM^LPS^ enhanced NF-κB p65 phosphorylation in 266-6 cells (Figure 6F), supporting the hypothesis that TNF-α might regulate SphK1 transcription through the NF-κB pathway.

To validate this mechanism, CM from RAW264.7 cells pretreated with CM from CCK-stimulated 266-6 cells (CM^CM-CCK^) or from untreated 266-6 cells (CM^CM-266-6^) was cocultured with 266-6 cells (Figure 6G). Notably, CM^CM-CCK^ treatment resulted in a marked upregulation of SphK1, along with enhanced activation of NF-κB signaling in 266-6 cells (Figure 6H).

Furthermore, CHIP assays confirmed obviously enrichment of NF-κB at two binding sites (site1 and site2) within the SphK1 promoter in 266-6 cells. This enrichment was further enhanced by CM^CM-CCK^ treatment but abrogated when NF-κB knockdown (Figure 6I). Consistently, CM^CM-CCK^ markedly enhanced luciferase activity in 266-6 cells transfected with WT SphK1 promoter reporter, an effect that was reversed by siNF-κB. In contrast, neither CM^CM-CCK^ nor siNF-κB influenced luciferase activity in 266-6 cells transfected with the mutant reporters lacking NF-κB binding sites (MUT1 and MU2) (Figure 6J). Both RT-qPCR and Western Blot assays showed that SphK1 expression was significantly reduced in CM^CM-CCK^-treated 266-6 cells following siNF-κB transfection (Figure 6K).

Collectively, these findings demonstrated that TNF-α/NF-κB pathway mediates the crosstalk between M1-polarized macrophages and iPACs by regulating SphK1 transcription.

### Inhibiting the SphK1/S1P/S1PR2 pathway impedes M1 macrophage polarization and alleviates inflammation in AP mice

**Figure 7:**
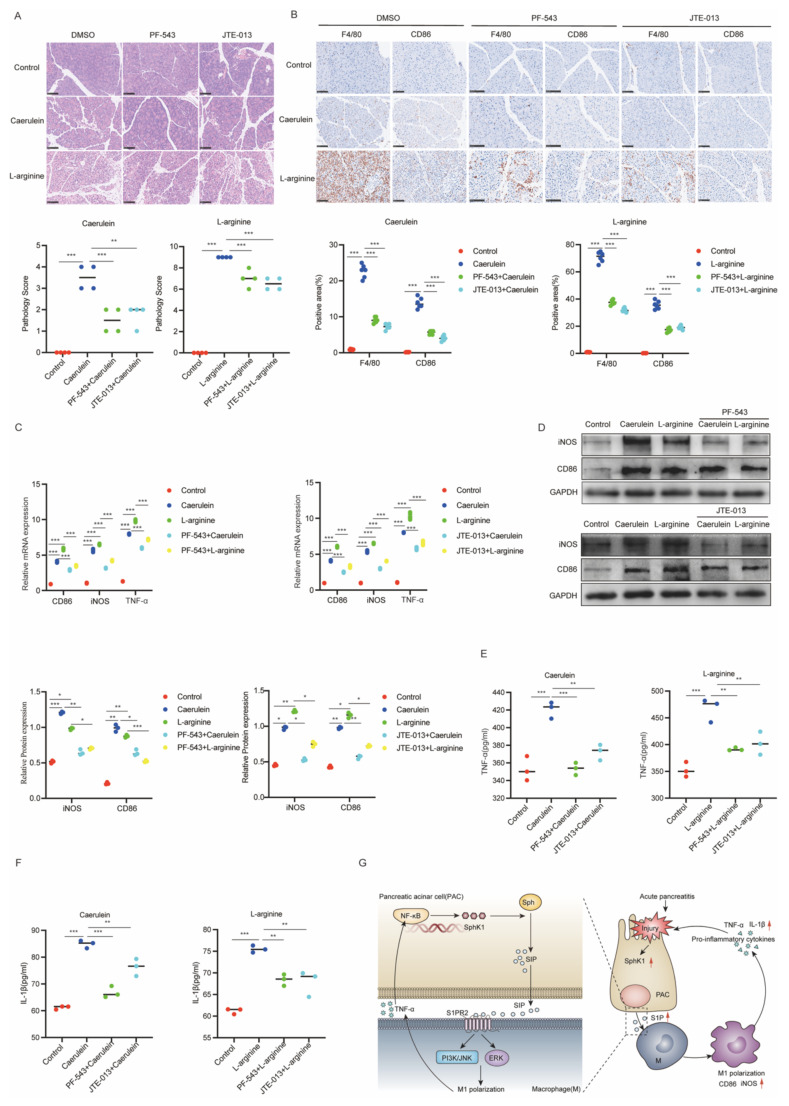
** Inhibiting the SphK1/S1P/S1PR2 pathway impedes M1 macrophage polarization and alleviates inflammation in AP mice.** The activity of SphK1 and S1PR2 of AP mice was inhibited by intraperitoneal injection of PF-543 and JTE-013 respectively. (A) Representative images of H&E staining in the pancreatic tissues of AP mice (n=4). Scale bar: 100 μm. (B) The expression of F4/80 and CD86 in the pancreatic tissues of AP mice were evaluated by IHC (n=6). Scale bar: 100 μm. (C) The mRNA levels of CD86, iNOS, and TNF-α in the pancreatic tissues of AP mice were determined by RT-qPCR. (D) The protein levels of CD86 and iNOS in the pancreatic tissues of AP mice were analyzed by Western blot. (E-F) The serum levels of TNF-α and IL-1β were measured by ELISA in AP mice. (G) Schematic diagram of iPACs induced M1 polarization of macrophage via SphK1/S1P signaling during AP. *P <0.05; **P <0.01; ***P <0.001; NS, no significance.

To further elucidate the role of the SphK1/S1P/S1PR2 pathway in AP, we selectively inhibited SphK1 and S1PR2 using the specific inhibitors PF-543 and JTE-013, respectively, and assessed their effects on inflammation and M1 polarization in AP mice. Treatment with either PF-543 or JTE-013 markedly alleviated pancreatic inflammation (Figure 7A) and inhibited M1 macrophage polarization, as evidenced by decreased F4/80 and CD86 in IHC staining (Figure 7B). Serum biochemistry analysis showed a marked decrease in amylase and lipase levels, suggesting improved pancreatic function (Supplementary Figure 4A and 4B). Notably, PF-543 treatment also significantly decreased serum S1P levels (Supplementary Figure 4C). Furthermore, both inhibitors effectively suppressed the expression of key M1 polarization markers (CD86, iNOS and TNF-α) in pancreatic tissue (Figure 7C and 7D) and reduced systemic pro-inflammatory cytokines, including TNF-α and IL-1β (Figure 7E and 7F). Collectively, these findings indicated that inhibition of the SphK1/S1P/S1PR2 pathway attenuates inflammation and M1 macrophage polarization in AP. A schematic representation illustrating iPACs-induced M1 polarization via the SphK1/S1P/S1PR2 axis is shown in Figure 7G.

## Discussion

Crosstalk between PACs and macrophages is increasingly recognized as a key driver of AP. Excessive mitochondrial reactive oxygen species (ROS) production in PACs induces mitochondrial dysfunction, leading to mitochondrial DNA (mtDNA) release and PAC apoptosis, mtDNA activates the cyclic GMP-AMP synthase-stimulator of interferon genes (cGAS-STING) signaling pathway in macrophages, thereby amplifying inflammatory responses and contributing to AP progression. Recent studies have shown that targeting mitochondrial ROS and mtDNA-triggered cGAS-STING signaling using nanomedicine effectively alleviates AP^36^, ^37^. In parallel, macrophage polarization critically influences disease severity, as excessive M1 polarization aggravates pancreatic tissue injury^26^. However, how iPACs regulate macrophage polarization during AP remains incompletely understood. To our knowledge, this study demonstrates a previously unrecognized role of the iPACs derived SphK1-S1P axis in promoting AP progression through the induction of macrophage M1 polarization.

The SphK1/S1P signaling pathway has been implicated in macrophage regulation in multiple inflammatory conditions^38^, and elevated SphK1 activity has been reported in peripheral immune cells from patients with early-stage SAP^23^. In this study, we observed increased SphK1 expression and S1P production in iPACs induced by AP *in vivo* and by CCK stimulation *in vitro*. Concomitantly, macrophage M1 polarization was enhanced in AP pancreatic tissue, whereas this response was attenuated in SphK1^-/-^ mice. *In vitro*, conditioned medium from CCK-stimulated 266-6 cells promoted M1 polarization, which was reversed by the SphK1 inhibitor PF543. Although 266-6 cells do not fully recapitulate primary PAC biology, these findings were further validated in primary PACs. Moreover, PF543 treatment alleviated inflammation in AP mice. Collectively, these findings suggest that the SphK1-mediated crosstalk between iPACs and macrophage M1 polarization plays an important role in the pathogenesis of AP.

S1P is a pleiotropic lipid mediator involved in immune regulation and endothelial function^39^-^42^. Discrepancies in circulating S1P levels reported in AP across different studies may be attributable to differences in disease severity and sampling time points. Previous reports describing reduced S1P levels focused mainly on patients with established severe AP^43^. In contrast, in the present study, plasma S1P levels were elevated in AP patients and correlated with disease severity, which may reflect disease stage-dependent regulation, as our samples were obtained predominantly during the early inflammatory phase. Consistently, exogenous S1P promoted M1 polarization in both RAW264.7 and BMDMs. These findings support the involvement of the SphK1/S1P axis in macrophage M1 polarization during AP.

S1PRs play a critical role in inflammatory diseases, including liver injury, sepsis, acute vascular inflammation, and AP^44^-^47^. S1PR2 mediates early pancreatic and systemic inflammatory responses via NF-κB activation and contributes to AP progression^47^. However, prior reports focused mainly on receptor-level signaling in PACs and did not address the upstream source of S1P. Here, we identify SphK1 activation in PACs as an upstream driver of S1P production that acts in a paracrine manner to regulate macrophage polarization during AP, whereas M1-polarized macrophages, in turn, enhance SphK1 expression in PACs through the TNF-α/NF-κB pathway. Coincidence with prior reports demonstrating that S1P-S1PR signaling influence immune cell polarization^48^, our data showed that S1PR2 was the most prominently upregulated S1PR in pancreatic tissue from AP mice and in macrophages stimulated with S1P or LPS. Notably, pharmacological inhibition of S1PR2 with JTE-013 markedly attenuated iPAC-induced M1 macrophage polarization and significantly reduced inflammatory responses in AP mice. We acknowledge that pharmacological inhibitors may exert off-target effects. To minimize this limitation, we employed concentrations commonly reported to achieve selective blockade. Importantly, the inhibitor-based observations were further corroborated by complementary genetic approaches, including siRNA-mediated knockdown of SphK1 and S1PR2, which produced consistent results. These findings suggest that the interaction between iPACs and macrophages is specifically mediated by the SphK1/S1P/ S1PR2 axis.

Previous studies have demonstrated that the PI3K and ERK signaling pathways play important roles in innate immune responses^49^-^52^. Additionally, it has been reported that S1P binding to S1PR2 or S1PR3 promotes M1 polarization of BMDM via the PI3K/JNK pathway in inflammatory liver disease^53^. Given this, we hypothesized that S1P might induce macrophages M1 polarization through the PI3K/JNK and ERK signaling pathways. Our results showed that the phosphorylation levels of PI3K, JNK and ERK were markedly increase in pancreatic tissue from AP mice. Consistently, macrophages treated with CM^CCK^ from iPACs, S1P, or LPS exhibited significantly elevated phosphorylation of PI3K, JNK and ERK. Furthermore, JTE-013 attenuated S1P-induced activation of these signaling pathways. These findings suggest that iPACs-derived S1P induces macrophages M1 polarization potentially through activation of PI3K/JNK and ERK signaling pathways.

Pro-inflammatory cytokines released from M1 macrophages are known to exacerbate pancreatic tissue and promote the activation of damage-related molecular patterns (DAMPs) in PACs^54^. Consistent with this notion, our study showed that PACs co-cultured with CM from S1P- or LPS-treated macrophages (CM^S1P^, CM^LPS^) exhibited significant cellular injury. Interestingly, exposure to CM^S1P^ or CM^LPS^ also increased SphK1 expression in these PACs. These results suggest the presence of a potential feedback loop between M1-polarized macrophages and PACs. Given that NF-κB is a central downstream transcription factor activated by diverse inflammatory signals and is critically involved in AP pathogenesis^55^, ^56^, we further examined its potential role. We found that the pro-inflammatory cytokines TNF-α and IL-1β significantly increased SphK1 mRNA expression in PACs. More importantly, activation of NF-κB was observed in PACs treated with CM^S1P^, CM^LPS^, or CM from macrophages co-cultured with CM^CCK^ derived from CCK-treated PACs. Furthermore, luciferase reporter and ChIP assays indicated that SphK1 can be transcriptionally regulated by NF-κB, this effect was enhanced by CM^CM-CCK^ and attenuated by siNF-κB. These findings suggest that inflammatory cytokines derived from M1 macrophages may promote SphK1 expression in PACs through NF-κB signaling, thereby supporting the existence of a positive inflammatory feedback loop during AP.

Emerging evidence suggests that the SphK1/S1P signaling pathway does not function in isolation but instead interacts with multiple inflammatory signaling networks, including NF-κB and MAPK pathways, which are central regulators of inflammatory responses in AP^57^. Previous studies have reported that S1PR2 is the predominant S1PR expressed in PACs and mediates NF-κB activation and early inflammatory responses during AP primary through ROCK-dependent signaling, rather than through extracellular signal-regulated kinase or p38 MAPK pathways^47^. In AP, coordinated activation of these signaling cascades may contribute to the establishment of inflammatory amplification loops that exacerbate pancreatic injury and accelerate disease progression. Together with prior reports, our findings support the concept that the SphK1/S1P axis may function as an upstream regulatory node within the inflammatory network in AP. Consequently, targeting this pathway may attenuate AP progression not only by directly affecting SphK1/S1P signaling but also by indirectly suppressing downstream inflammatory pathways.

Collectively, our findings highlight a reciprocal feedback loop between iPACs and M1 macrophages contributes to the progression of AP. Although therapeutic targeting of this pathway is conceptually attractive, its clinical translation will require careful consideration. Future studies should focus on delineating cell-type-specific functions of SphK1-S1P signaling, defining the optimal timing of intervention within the narrow therapeutic window of AP, and developing strategies to enhance target specificity while minimizing systemic effects. In addition, advanced drug-delivery approaches may facilitate selective modulation of this pathway within the inflamed pancreas. Addressing these challenges will be essential for determining whether modulation of the SphK1-S1P axis can be translated into a safe and effective therapeutic approach for AP.

## Supplementary Material

Supplementary figures and tables.

## Data Availability

Data are available upon reasonable request.

## Abbreviations

AP: acute pancreatitis
CCK: cholecystokinin
CM: conditioned media
iPAC: injured pancreatic acinar cell
p-: phosphorylated
PCR: polymerase chain reaction
RT: reverse-transcription
si: small interfering
S1P: sphingosine-1-phosphate
S1PR: sphingosine-1-phosphate receptor
SphK1: sphingosine kinase 1
WB: western blot
WT: wild-type

## References

[1] Gukovskaya AS, Pandol SJ, Gukovsky I (2016). New insights into the pathways initiating and driving pancreatitis. Curr Opin Gastroen.

[2] Lugea A, Waldron RT, Mareninova OA, Shalbueva N, Deng N, Su HY (2017). Human Pancreatic Acinar Cells: Proteomic Characterization, Physiologic Responses, and Organellar Disorders in ex Vivo Pancreatitis. Am J Pathol.

[3] Xiao AY, Tan ML, Wu LM, Asrani VM, Windsor JA, Yadav D (2016). Global incidence and mortality of pancreatic diseases: a systematic review, meta-analysis, and meta-regression of population-based cohort studies. Lancet Gastroenterol Hepatol.

[4] Schepers NJ, Bakker OJ, Besselink MG, Ali UA, Bollen TL, Gooszen HG (2019). Impact of characteristics of organ failure and infected necrosis on mortality in necrotising pancreatitis. Gut.

[5] Bang JY, Wilcox CM, Arnoletti JP, Varadarajulu S (2020). Superiority of endoscopic interventions over minimally invasive surgery for infected necrotizing pancreatitis: meta-analysis of randomized trials. Dig Endosc.

[6] Gurusamy KS, Belgaumkar AP, Haswell A, Pereira SP, Davidson BR (2016). Interventions for necrotising pancreatitis. Cochrane Database Syst Rev.

[7] van Santvoort HC, Bakker OJ, Bollen TL, Besselink MG, Ahmed Ali U, Schrijver AM (2011). A conservative and minimally invasive approach to necrotizing pancreatitis improves outcome. Gastroenterology.

[8] Lankisch PG, Apte M, Banks PA (2015). Acute pancreatitis. Lancet.

[9] Mayerle J (2009). A novel role for leucocytes in determining the severity of acute pancreatitis. Gut.

[10] Habtezion A (2015). Inflammation in acute and chronic pancreatitis. Curr Opin Gastroenterol.

[11] Saeki K, Kanai T, Nakano M, Nakamura Y, Miyata N, Sujino T (2012). CCL2-Induced Migration and SOCS3-Mediated Activation of Macrophages Are Involved in Cerulein-Induced Pancreatitis in Mice. Gastroenterology.

[12] Watanabe S, Alexander M, Misharin AV, Budinger GRS (2019). The role of macrophages in the resolution of inflammation. J Clin Invest.

[13] Wynn TA, Vannella KM (2016). Macrophages in Tissue Repair, Regeneration, and Fibrosis. Immunity.

[14] Murray PJ, Allen JE, Biswas SK, Fisher EA, Gilroy DW, Goerdt S (2014). Macrophage activation and polarization: nomenclature and experimental guidelines. Immunity.

[15] Shrivastava P, Bhatia M (2010). Essential role of monocytes and macrophages in the progression of acute pancreatitis. World J Gastroentero.

[16] Gea-Sorli S, Closa D (2010). Role of macrophages in the progression of acute pancreatitis. World J Gastrointest Pharmacol Ther.

[17] Sendler M, Weiss FU, Golchert J, Homuth G, van den Brandt C, Mahajan UM (2018). Cathepsin B-Mediated Activation of Trypsinogen in Endocytosing Macrophages Increases Severity of Pancreatitis in Mice. Gastroenterology.

[18] Olivera A, Kohama T, Tu Z, Milstien S, Spiegel S (1998). Purification and characterization of rat kidney sphingosine kinase. J Biol Chem.

[19] Sanchez T, Hla T (2004). Structural and functional characteristics of S1P receptors. J Cell Biochem.

[20] Rothhammer V, Kenison JE, Tjon E, Takenaka MC, de Lima KA, Borucki DM (2017). Sphingosine 1-phosphate receptor modulation suppresses pathogenic astrocyte activation and chronic progressive CNS inflammation. Proc Natl Acad Sci U S A.

[21] Feng A, Rice AD, Zhang Y, Kelly GT, Zhou T, Wang T (2020). S1PR1-Associated Molecular Signature Predicts Survival in Patients with Sepsis. Shock.

[22] Bamias G, Rivera-Nieves J (2016). Targeting S1P Receptors, A New Mechanism of Action for Inflammatory Bowel Disease Therapy. Gastroenterology.

[23] Li Q, Wang C, Zhang Q, Tang C, Li N, Li J (2012). The role of sphingosine kinase 1 in patients with severe acute pancreatitis. Ann Surg.

[24] Wang D, Han S, Lv G, Hu Y, Zhuo W, Zeng Z (2023). Pancreatic Acinar Cells-Derived Sphingosine-1-Phosphate Contributes to Fibrosis of Chronic Pancreatitis via Inducing Autophagy and Activation of Pancreatic Stellate Cells. Gastroenterology.

[25] Spormann H, Sokolowski A, Letko G (1989). Effect of temporary ischemia upon development and histological patterns of acute pancreatitis in the rat. Pathol Res Pract.

[26] Peng C, Tu G, Wang J, Wang Y, Wu P, Yu L (2023). MLKL signaling regulates macrophage polarization in acute pancreatitis through CXCL10. Cell Death Dis.

[27] Zhao Q, Wei Y, Pandol SJ, Li L, Habtezion A (2018). STING Signaling Promotes Inflammation in Experimental Acute Pancreatitis. Gastroenterology.

[28] Tian J, Chang S, Wang J, Chen J, Xu H, Huang T (2023). S1P/S1PR1 axis promotes macrophage M1 polarization through NLRP3 inflammasome activation in Lupus nephritis. Molecular Immunology.

[29] Gong L, Shen Y, Wang S, Wang X, Ji H, Wu X (2023). Nuclear SPHK2/S1P induces oxidative stress and NLRP3 inflammasome activation via promoting p53 acetylation in lipopolysaccharide-induced acute lung injury. Cell Death Discovery.

[30] Drexler Y, Molina J, Mitrofanova A, Fornoni A, Merscher S (2021). Sphingosine-1-Phosphate Metabolism and Signaling in Kidney Diseases. J Am Soc Nephrol.

[31] Ren K, Lu Y-J, Mo Z-C, Liu X, Tang Z-L, Jiang Y (2017). ApoA-I/SR-BI modulates S1P/S1PR2-mediated inflammation through the PI3K/Akt signaling pathway in HUVECs. Journal of Physiology and Biochemistry.

[32] Li S, Chen J, Fang X, Xia X (2017). Sphingosine-1-phosphate activates the AKT pathway to inhibit chemotherapy induced human granulosa cell apoptosis. Gynecological Endocrinology.

[33] Kerneur C, Cano CE, Olive D (2022). Major pathways involved in macrophage polarization in cancer. Front Immunol.

[34] Vergadi E, Ieronymaki E, Lyroni K, Vaporidi K, Tsatsanis C (2017). Akt Signaling Pathway in Macrophage Activation and M1/M2 Polarization. The Journal of Immunology.

[35] Pan HY, Yang H, Shao MY, Xu J, Zhang P, Cheng R (2014). Sphingosine-1-phosphate mediates AKT/ERK maintenance of dental pulp homoeostasis. International Endodontic Journal.

[36] Liu J, Wang D, Wang S, Shi X, Xiong T, Lu Y (2026). Dual-targeted molybdenum nanomedicine treats acute pancreatitis by blocking mitochondrial DNA-triggered cGAS-STING signaling via ROS modulation. J Control Release.

[37] Wang D, Wang S, Liu J, Shi X, Xiong T, Li R (2025). Nanomedicine Penetrating Blood-Pancreas Barrier for Effective Treatment of Acute Pancreatitis. Adv Sci (Weinh).

[38] Padmam Puneet CTY, Lingkai Wong, Lam Yulin, Dow Rhoon Koh, Shabbir Moochhala, Josef Pfeilschifter, Andrea Huwiler, Alirio J (2010). Melendez. SphK1 Regulates Proinflammatory Responses Associated with Endotoxin and Polymicrobial Sepsis. Science.

[39] Spiegel S, Milstien S (2011). The outs and the ins of sphingosine-1-phosphate in immunity. Nat Rev Immunol.

[40] Lee MJ, Van Brocklyn JR, Thangada S, Liu CH, Hand AR, Menzeleev R (1998). Sphingosine-1-phosphate as a ligand for the G protein coupled receptor EDG-1. Science.

[41] Hla T, Dannenberg AJ (2012). Sphingolipid signaling in metabolic disorders. Cell Metab.

[42] Cartier A, Hla T (2019). Sphingosine 1-phosphate: Lipid signaling in pathology and therapy. Science.

[43] Wollny T, Watek M, Wnorowska U, Piktel E, Gozdz S, Kurek K (2022). Hypogelsolinemia and Decrease in Blood Plasma Sphingosine-1-Phosphate in Patients Diagnosed with Severe Acute Pancreatitis. Dig Dis Sci.

[44] Wang Y, Aoki H, Yang J, Peng K, Liu R, Li X (2017). The role of sphingosine 1-phosphate receptor 2 in bile-acid-induced cholangiocyte proliferation and cholestasis-induced liver injury in mice. Hepatology.

[45] Chen L, Li L, Song Y, Lv T (2021). Blocking SphK1/S1P/S1PR1 Signaling Pathway Alleviates Lung Injury Caused by Sepsis in Acute Ethanol Intoxication Mice. Inflammation.

[46] Zhang G, Yang L, Kim GS, Ryan K, Lu S, O'Donnell RK (2013). Critical role of sphingosine-1-phosphate receptor 2 (S1PR2) in acute vascular inflammation. Blood.

[47] Yang J, Tang X, Li B, Shi J (2022). Sphingosine 1-phosphate receptor 2 mediated early stages of pancreatic and systemic inflammatory responses via NF-kappa B activation in acute pancreatitis. Cell Commun Signal.

[48] Tiper IV, East JE, Subrahmanyam PB, Webb TJ (2016). Sphingosine 1-phosphate signaling impacts lymphocyte migration, inflammation and infection. Pathog Dis.

[49] Koyasu S (2003). The role of PI3K in immune cells. Nat Immunol.

[50] Lucas CL, Chandra A, Nejentsev S, Condliffe AM, Okkenhaug K (2016). PI3Kδ and primary immunodeficiencies. Nat Rev Immunol.

[51] Arthur JS, Ley SC (2013). Mitogen-activated protein kinases in innate immunity. Nat Rev Immunol.

[52] Xu J, Hopkins K, Sabin L, Yasunaga A, Subramanian H, Lamborn I (2013). ERK signaling couples nutrient status to antiviral defense in the insect gut. Proc Natl Acad Sci U S A.

[53] Yang J, Yang L, Tian L, Ji X, Yang L, Li L (2018). Sphingosine 1-Phosphate (S1P)/S1P Receptor2/3 Axis Promotes Inflammatory M1 Polarization of Bone Marrow-Derived Monocyte/Macrophage via G(alpha)i/o/PI3K/JNK Pathway. Cell Physiol Biochem.

[54] Hu F, Lou N, Jiao J, Guo F, Xiang H, Shang D (2020). Macrophages in pancreatitis: Mechanisms and therapeutic potential. Biomed Pharmacother.

[55] Rakonczay Z Jr, Hegyi P, Takacs T, McCarroll J, Saluja AK (2008). The role of NF-kappaB activation in the pathogenesis of acute pancreatitis. Gut.

[56] Jakkampudi A, Jangala R, Reddy BR, Mitnala S, Nageshwar Reddy D, Talukdar R (2016). NF-kappaB in acute pancreatitis: Mechanisms and therapeutic potential. Pancreatology.

[57] Fu F, Li W, Zheng X, Wu Y, Du D, Han C (2024). Role of Sphingosine-1-Phosphate Signaling Pathway in Pancreatic Diseases. Int J Mol Sci.

