# Supplementary material for: SphK1/S1P signaling-mediated crosstalk between pancreatic acinar cell and macrophage M1 polarization aggravates acute pancreatitis progression
Source: Int J Biol Sci. 2026 Mar 25;22(7):3826–42. doi: 10.7150/ijbs.120627 (PMC13086089; doi:10.7150/ijbs.120627)
Supplement: Supplementary file 1 — Supplementary figures and tables. [file ijbsv22p3826s1.pdf]

Supplementary Figures

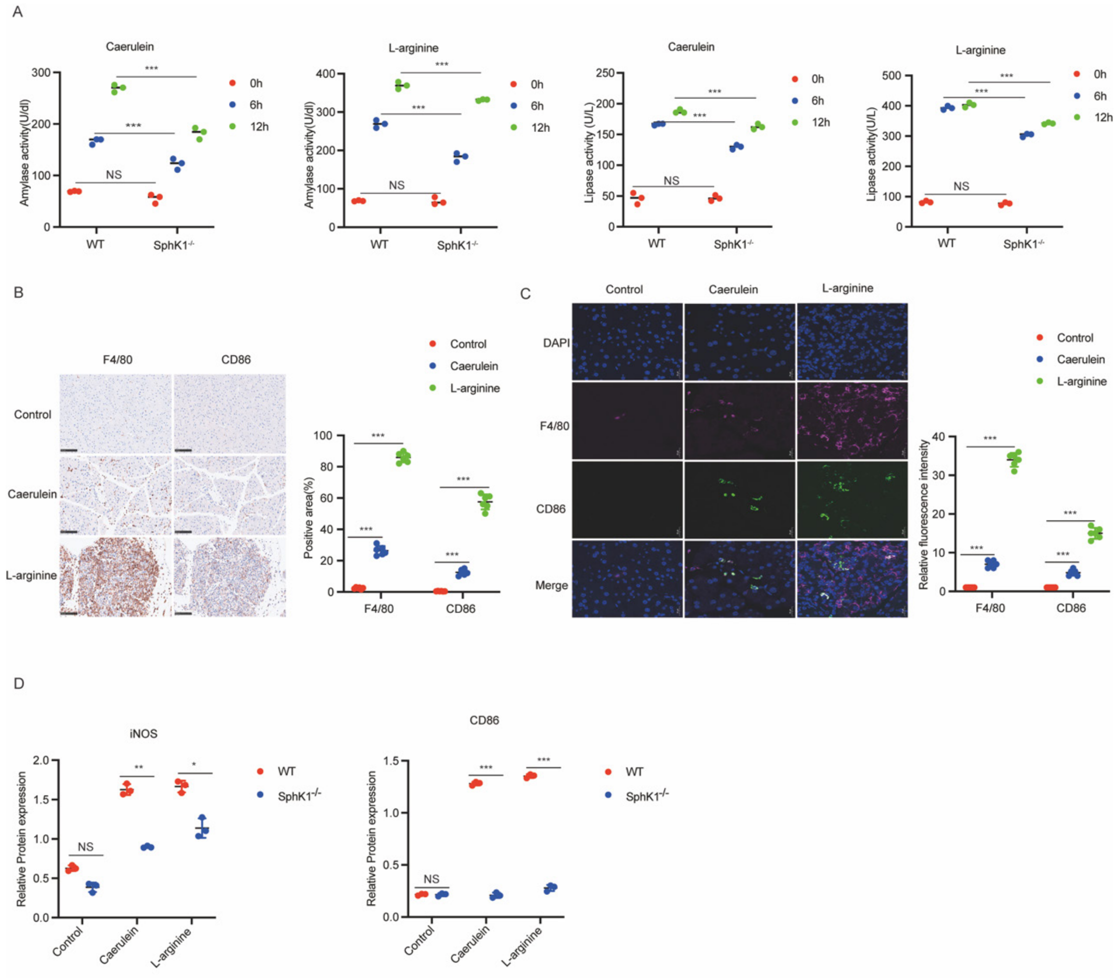

Supplementary Figure 1

(A) Serum AMY and LPS levels in WT and SphK1$^{-/-}$ mice with AP. (B) F4/80 and CD86 expression in pancreatic tissues of WT AP mice evaluated by IHC (n=6). Scale bar: 100 μm. (C) F4/80 and CD86 expression in the pancreatic tissues of WT AP mice evaluated by IF (n=6). Scale bar: 20 μm. (D) Relative protein expression of CD86 and iNOS in the pancreatic tissues from WT and SphK1$^{-/-}$ AP mice. *P <0.05; **P <0.01; ***P <0.001; NS, no significance.

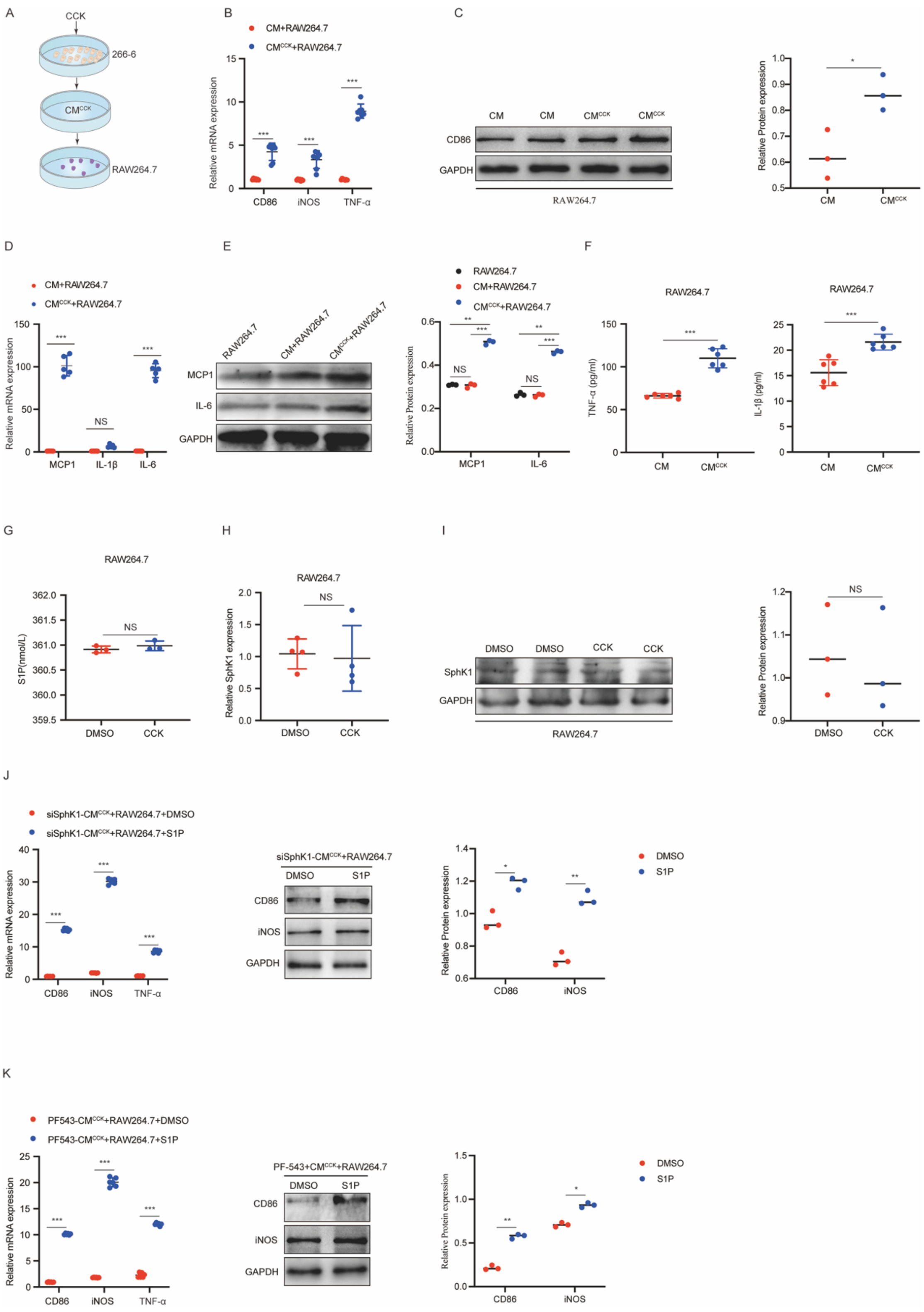

Supplementary Figure 2

(A) Schematic of the co-culture system using CM^{CCK} and RAW264.7 cells. (B) The mRNA level of CD86, iNOS and TNF-α in RAW264.7 cells co-cultured with CM^{CCK}.

(C) The protein level of CD86 in RAW264.7 cells co-cultured with CM^{CCK}. (D-E) The expression of MCP1, IL-1β and IL-6 in RAW264.7 cells co-cultured with CM^{CCK}. (F) The level of TNF-α and IL-1β in the supernatant of RAW264.7 cells co-cultured with CM^{CCK}. (G) S1P level in the supernatant of CCK-treated RAW264.7 cells. (H-I) The expression of SphK1 in CCK-treated RAW264.7 cells. (J-K) Effect of exogenous S1P on CD86, iNOS, and TNF-α mRNA and the protein levels in RAW264.7 cells co-cultured with siSphK1-CM^{CCK} or PF543-CM^{CCK}. *P <0.05; **P <0.01; ***P <0.001; NS, no significance.

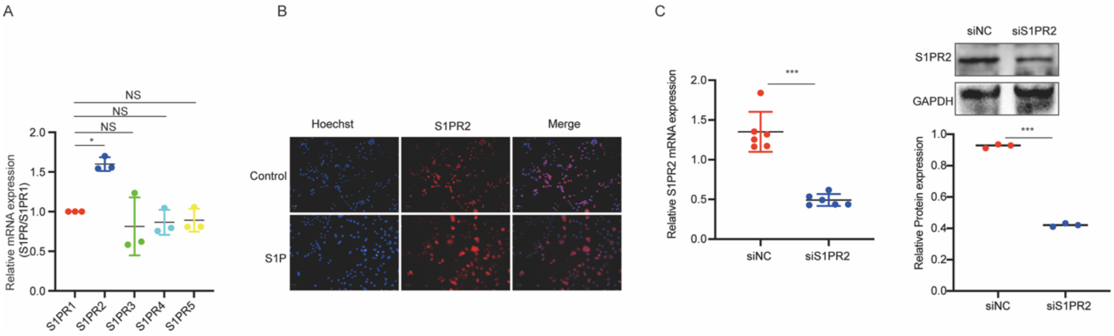

Supplementary Figure 3

(A) Among all SIPRs, S1PR2 mRNA expression was significantly increased in S1P-treated RAW264.7 cells. (B) IF images of S1PR2 (in red) in S1P-treated RAW264.7 cells. Nuclei were counterstained with DAPI (in blue). Scale bar: 20 μm. (C) S1PR2 was inhibited by siS1PR2. *P <0.05; **P <0.01; ***P <0.001; NS, no significance.

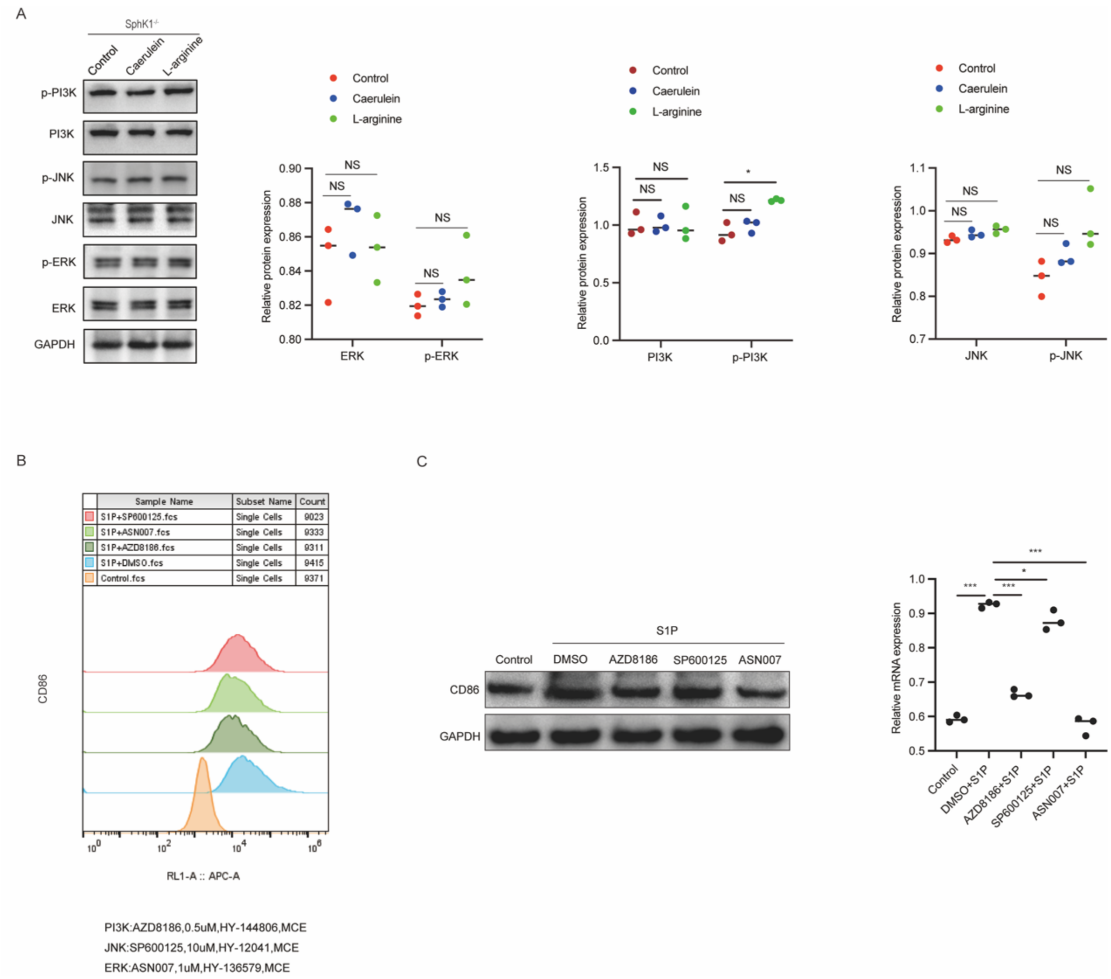

Supplementary Figure 4

(A) The protein levels of PI3K, JNK and ERK in pancreatic tissues from SphK1$^{-/-}$ AP mice were analyzed by Western blot. (B) After inhibition of the PI3K/JNK and ERK pathways, the percentage of CD86-positive RAW264.7 cells treated with S1P was assessed by flow cytometry. (C) After blocking the PI3K/JNK and ERK pathways, CD86 protein levels were examined by western blot in S1P-treated RAW 264.7 cells. *P <0.05; **P <0.01; ***P <0.001; NS, no significance.

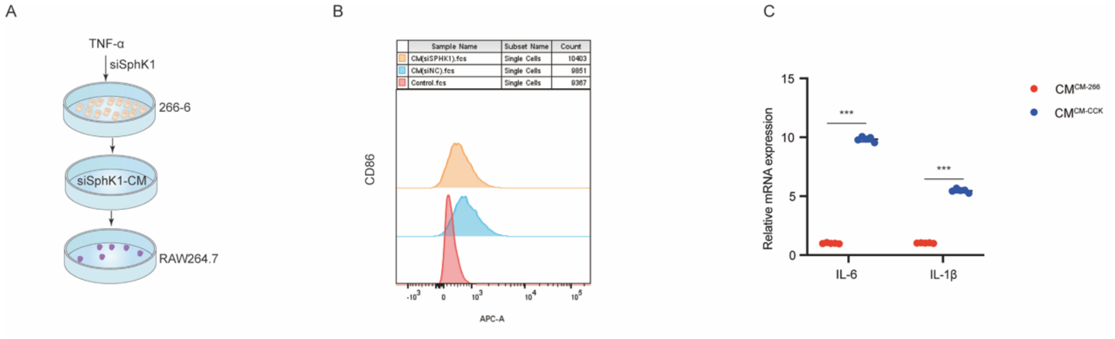

Supplementary Figure 5

(A) Under TNF-α pretreatment, SphK1 was simultaneously knocked out in 266-6 cells, which were then co-cultured with RAW264.7 cells. (B) The percentage of CD86-positive RAW264.7 cells treated with CM^{siSphK1} was assessed by flow cytometry. (C) mRNA levels of downstream targets of the NF-κB signaling pathway were quantified by qPCR. *P <0.05; **P <0.01; ***P <0.001; NS, no significance.

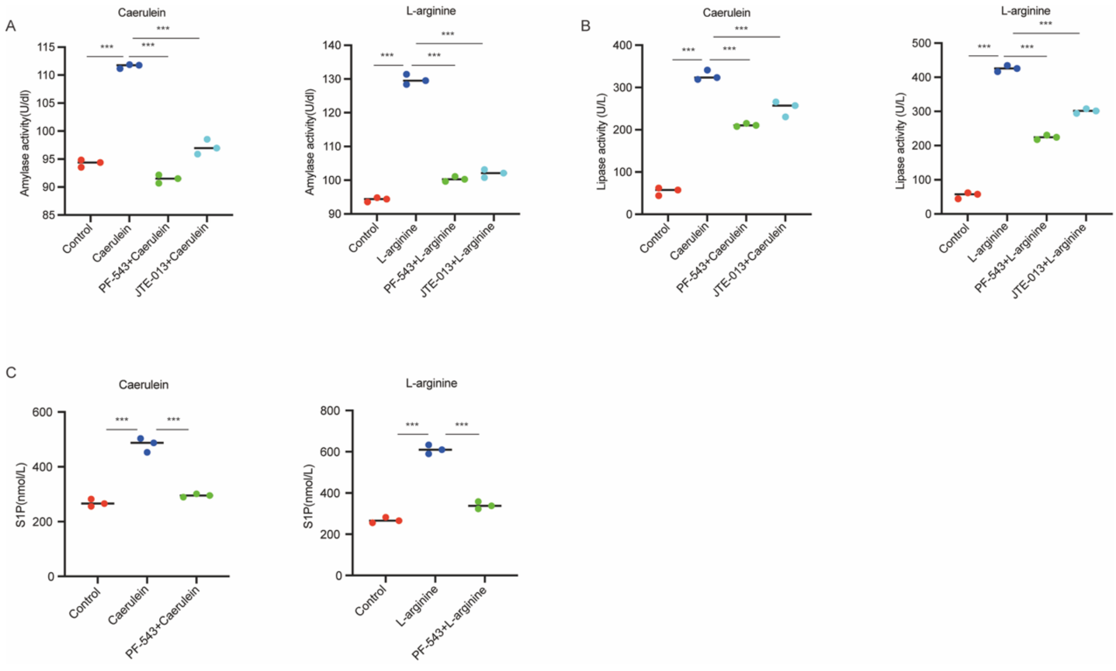

Supplementary Figure 6

(A-B) Pancreatic exocrine function was evaluated by measuring serum levels of AMY and LPS in AP mice. (C) S1P levels in mice serum were measured by ELISA. *P <0.05; **P <0.01; ***P <0.001; NS, no significance.

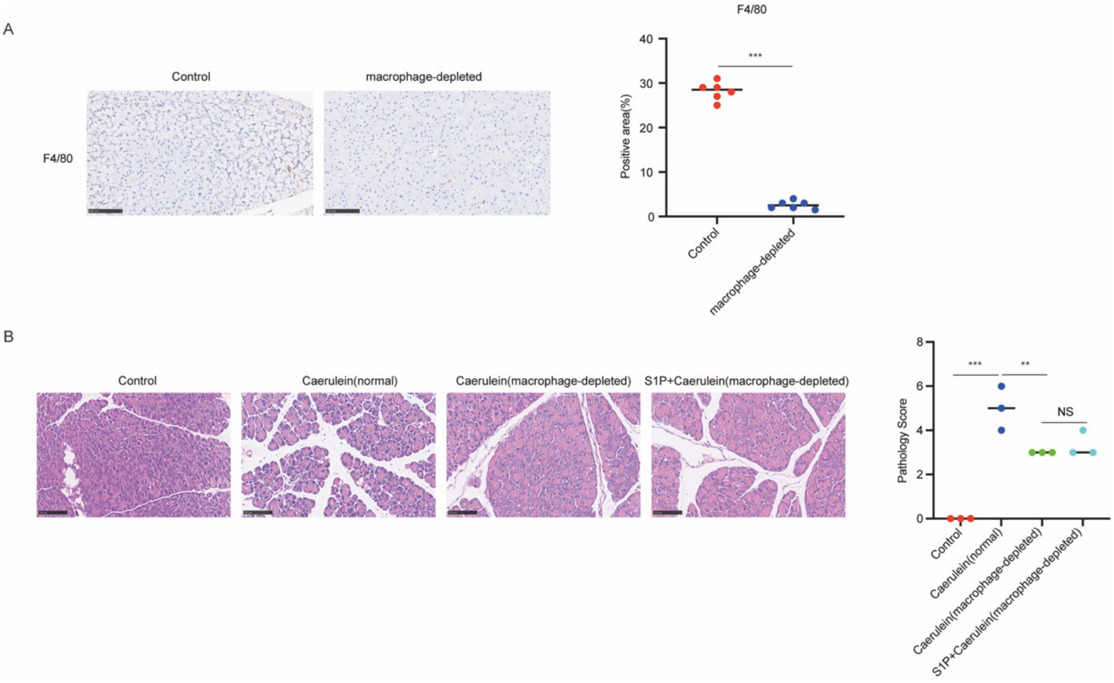

Supplementary Figure 7

(A) The expression of F4/80 in the pancreatic tissues from control and macrophage-depleted mice was evaluated by IHC (n=6). (B) Representative H&E staining sections of pancreatic tissues from AP mice following macrophage-depleted and S1P intervention. Quantification was performed in a pathology score manner (n=3). Scale bars, 100 μm. *P <0.05; **P <0.01; ***P <0.001; NS, no significance.

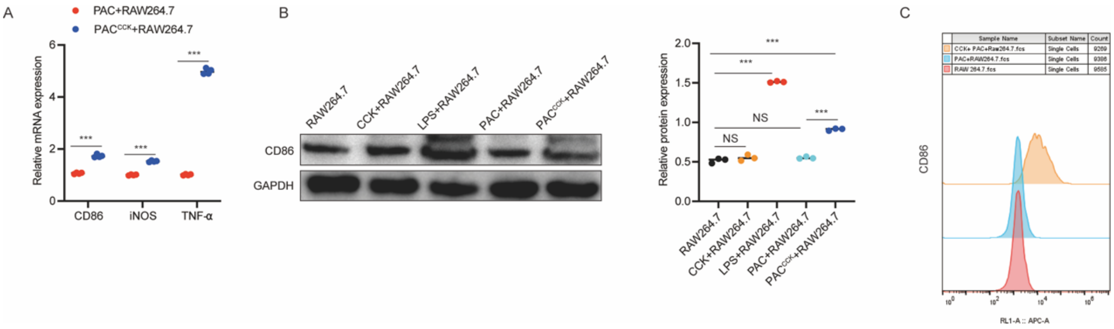

Supplementary Figure 8

(A) The mRNA levels of CD86, iNOS, and TNF-α in RAW264.7 cells co-cultured with CCK-injured PACs. (B) CD86 protein level in RAW264.7 cells co-cultured with CCK-injured PACs. (C) The percentage of CD86-positive RAW264.7 cells following co-cultured with CCK-injured PACs was assessed by flow cytometry. *P <0.05; **P <0.01; ***P <0.001; NS, no significance.

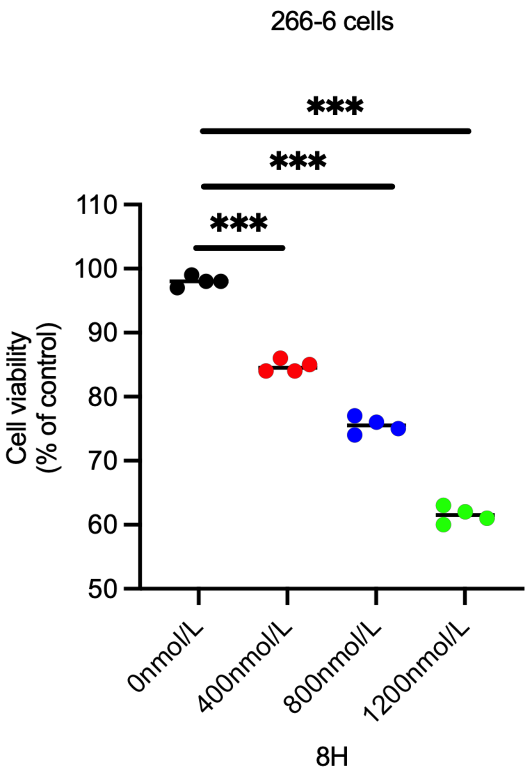

Supplementary Figure 9

Quantification of cell viability of 266–6 cells treated with various CCK concentrations for 8h. *P <0.05; **P <0.01; ***P <0.001; NS, no significance.

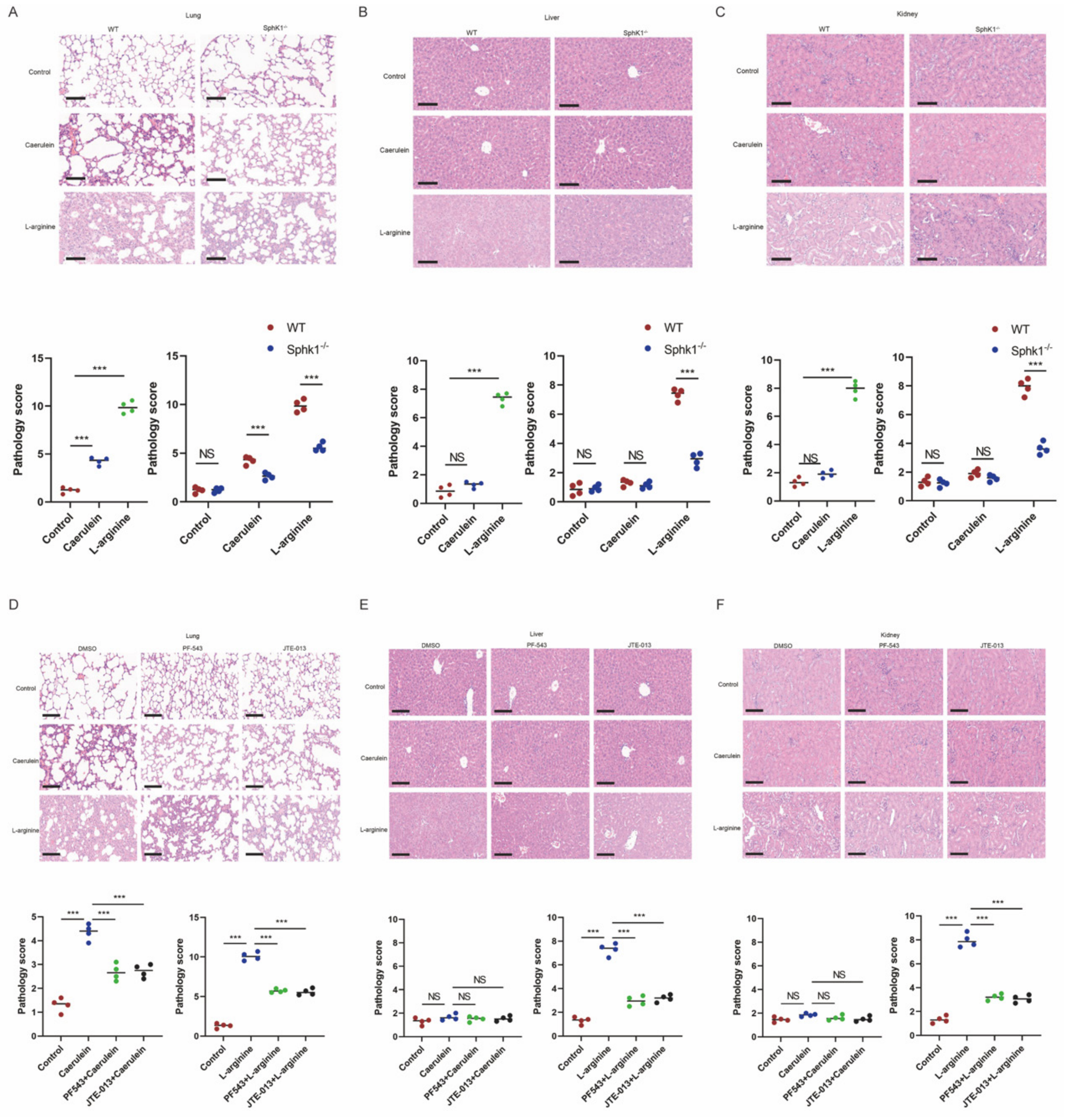

Supplementary Figure 10

(A-C) Representative images of H&E staining in the lung, liver and kidney from WT and SphK1$^{-/-}$ AP mice (n=4). (D-F) Representative images of H&E staining in the lung, liver and kidney from AP mice treated with PF-543 or JTE-013 (n=4). Scale bar: 50 µm. *P <0.05; **P <0.01; ***P <0.001; NS, no significance.

Table S1. The sequences for gene knockdown.

siRNA Targets	Sequences
SphK1-siRNA#1 sense	5′- GAGGCAGAGAUAACCUUUATT -3′
antisence	5′- UAAAGGUUAUCUCUGCCUCTT -3′
SphK1-siRNA#2 sense	5′- GGTGAATGGGCTAATGGAACG -3′
antisence	5′- CTGCTCGTACCCAGCATAGTG -3′
SphK1-siRNA#3 sense	5′- ATGGAACCAGTAGAATGCCCT -3′
antisence	5′- TCCGTTCGGTGAGTATCAGTTTA -3′
S1PR2-siRNA#1 sense	5′- CCGUCAUCUUACUGGCUAUTT -3′
antisence	5′- AUAGCCAGUAAGAUGACGGTT -3′
S1PR2-siRNA#2 sense	5′- GCUCUCCGCCUCGGUCUUUTT -3′
antisence	′5′- AAAGACCGAGGCGGAGAGCTT -3′
S1PR2-siRNA#3 sense	5′- GCCUACAUCACCGACAUUUTT -3′
antisence	5′- AAAUGUCGGUGAUGUAGGCTT -3′
NC-siRNA sense	5′- UUCUCCGAACGUGUCACGUTT -3′
antisence	5′- ACGUGACACGUUCGGAGAATT -3′

Table S2. The sequences of PCR primers.

Primer	Sequence
SphK1	Forward: 5′- GGTGAATGGGCTAATGGAACG -3′
	Reverse: 5′- CTGCTCGTACCCAGCATAGTG -3′

TNF-α	Forward: 5′- CCTGTAGCCCACGTCGTAG -3′
	Reverse: 5′- GGGAGTAGACAAGGTACAACC -3′
NOS2	Forward: 5′- GTTCTCAGCCCAACAATACAAGA -3′
	Reverse: 5′- GTGGACGGGTCGATGTCAC -3′
CD86	Forward: 5′- CTGGACTCTACGACTTCACAATG -3′
	Reverse: 5′- AGTTGGCGATCACTGACAGTT -3
S1PR1	Forward: 5’- ATGGTGTCCACTAGCATCCC -3’
	Reverse: 5’- CGATGTTCAACTTGCCTGTGTAG -3’
S1PR2	Forward: 5’- ACAGCAAGTTCCACTCAGCAA -3’
	Reverse: 5’- CTGCACGGGAGTTAAGGACAG -3’
S1PR3	Forward: 5’- ACTCTCCGGGAACATTACGAT -3’
	Reverse: 5’- CCAAGACGATGAAGCTACAGG -3’
S1PR4	Forward: 5′- GTCAGGGACTCGTACCTTCCA -3′
	Reverse: 5′- GATGCAGCCATACACACGG -3′
S1PR5	Forward: 5′- CCTGCTTCGTACCCTTAGCG -3′
	Reverse: 5′- GGCACGCGACATCCAGTAAT -3′
Arg1	Forward: 5′- CATATCTGCCAAAGACATCGTG -3′
	Reverse: 5′- GACATCAAAGCTCAGGTGAATC -3′
CD206	Forward: 5′- CCTATGAAAATTGGGCTTACGG -3′
	Reverse: 5′- CTGACAAATCCAGTTGTTGAGG -3′
IL-6	Forward: 5′- CTGCAAGAGACTTCCATCCAG -3′
	Reverse: 5′- AGTGGTATAGACAGGTCTGTTGG -3′

IL-1β	Forward: 5′- TTCAGGCAGGCAGTATCACTC -3′
	Reverse: 5′- GAAGGTCCACGGGAAAGACAC -3′
MCP1	Forward: 5′- TTAAAAACCTGGATCGGAACCAA-3′
	Reverse: 5′- GCATTAGCTTCAGATTTACGGGT -3′
ACTB	Forward: 5′- GTGACGTTGACATCCGTAAAGA -3′
	Reverse: 5′- GCCGGACTCATCGTACTCC -3′
